# A comparative transcriptional landscape of maize and sorghum obtained by single-molecule sequencing

**DOI:** 10.1101/gr.227462.117

**Published:** 2018-06

**Authors:** Bo Wang, Michael Regulski, Elizabeth Tseng, Andrew Olson, Sara Goodwin, W. Richard McCombie, Doreen Ware

**Affiliations:** 1 Cold Spring Harbor Laboratory, Cold Spring Harbor, New York 11724, USA;; 2 Pacific Biosciences, Menlo Park, California 94025, USA;; 3 USDA ARS NEA Robert W. Holley Center for Agriculture and Health, Cornell University, Ithaca, New York 14853, USA

## Abstract

Maize and sorghum are both important crops with similar overall plant architectures, but they have key differences, especially in regard to their inflorescences. To better understand these two organisms at the molecular level, we compared expression profiles of both protein-coding and noncoding transcripts in 11 matched tissues using single-molecule, long-read, deep RNA sequencing. This comparative analysis revealed large numbers of novel isoforms in both species. Evolutionarily young genes were likely to be generated in reproductive tissues and usually had fewer isoforms than old genes. We also observed similarities and differences in alternative splicing patterns and activities, both among tissues and between species. The maize subgenomes exhibited no bias in isoform generation; however, genes in the B genome were more highly expressed in pollen tissue, whereas genes in the A genome were more highly expressed in endosperm. We also identified a number of splicing events conserved between maize and sorghum. In addition, we generated comprehensive and high-resolution maps of poly(A) sites, revealing similarities and differences in mRNA cleavage between the two species. Overall, our results reveal considerable splicing and expression diversity between sorghum and maize, well beyond what was reported in previous studies, likely reflecting the differences in architecture between these two species.

Evolutionary developmental studies have been used to investigate the relationships between molecular characteristics and individual traits. Closely related species can exhibit huge morphological differences, despite sharing high genome sequence similarities. For example, genomic and transcriptomic comparisons between human and chimpanzee have identified molecular differences that may underlie some of the unique attributes of these primate species (Preuss et al. 2004; Bustamante et al. 2005; Khaitovich et al. 2006). These differences are the result of both transcript variations (Pollard et al. 2006; Prabhakar et al. 2006) and genomic structural variations (Cheng et al. 2005, Newman et al. 2005; Perry et al. 2006). In addition, a comparison of the transcriptional landscapes of human and mouse tissues revealed considerable RNA expression diversity between the two species that likely explains their fundamental physiological differences (Lin et al. 2014). Gene expression during development recapitulates the hourglass model (Domazet-Lošo and Tautz 2010; Quint et al. 2012; Cui et al. 2015), in which divergence is more extensive early and late in development than in the middle. In plants, conserved alternative splicing (AS) events have been identified between *Arabidopsis* and rice (Campbell et al. 2006), *Brassica* and *Arabidopsis* (Darracq and Adams 2013), and rice and maize (Severing et al. 2009). However, only a small fraction of the events conserved among species have been identified.

Maize and sorghum have very similar morphologies and phylogeny. This kinship is reflected by the fact that maize is an ancient tetraploid that underwent whole-genome duplication 5–12 Myr ago (Bomblies and Doebley 2005; Schnable et al. 2009), and its genome can be divided into two subgenomes, A and B, based on the most closely related unduplicated genome, sorghum (Schnable et al. 2011; Jiao et al. 2017). Despite the recent accumulation of data revealing gene expression patterns during development in maize (Sekhon et al. 2011; Stelpflug et al. 2016) and sorghum (Gelli et al. 2014; Shakoor et al. 2014), few studies have compared the transcriptome landscapes of the two species, especially in terms of differential AS, using matched tissues. Although RNA-seq has been widely applied in quantitative analyses, isoforms assembled from short-read sequencing are much less accurate than those assembled from single-molecule long-read sequencing (Wang et al. 2016). Multiple studies have demonstrated the power and reliability of long-read sequencing, especially for identification of full-length isoforms (Treutlein et al. 2014; Abdel-Ghany et al. 2016; Wang et al. 2016). In this study, we used RNA-seq and single-molecule long-read sequencing to compare transcriptome changes in matched tissues of maize and sorghum.

## Results

### Full-length sequencing and bioinformatics pipeline

We extracted high-quality (HQ) RNA from 11 matched tissues of maize B73 and sorghum BTx623 at different developmental stages for gene expression profiling. The RNA was used to generate size-fractionated libraries for single-molecule sequencing on Pacific Biosciences (PacBio) platforms, yielding 6,893,280 reads (Supplemental Table S1). Each size-selected library had the expected distribution of transcript lengths, ranging from 256–6643 bp (Supplemental Table S2); 45.5% of reads were classified as full length based on the presence of barcoded primers and poly(A) tails. ToFU processing yielded 1,624,076 full-length, HQ consensus transcript sequences. Sequencing length distributions were well matched to those of the corresponding size-fractionated libraries.

Mapping of HQ transcripts to reference genomes yielded 1,570,093 (96.7%) sequences that collapsed into 136,745 nonredundant isoforms for maize and 979,305 (89.5%) sequences that collapsed into 95,380 nonredundant isoforms for sorghum. Reads differing only at the 5′ start site within the first exon were considered redundant, and only the longest version was retained. We discarded 53,983 (3.3%) and 115,054 (10.5%) sequences due to low coverage or identity from maize and sorghum, respectively. Genome-wide BLASTX of these unmapped sequences to NCBI RefSeq proteins revealed that 54.3% (maize) and 44.6% (sorghum) of them fell into gaps in the assembly, whereas the remaining sequences could be mapped to other organisms and thus represent biological contaminants from endophytes or other sources.

### Isoform detection and characterization in maize and sorghum

**Figure 1.**
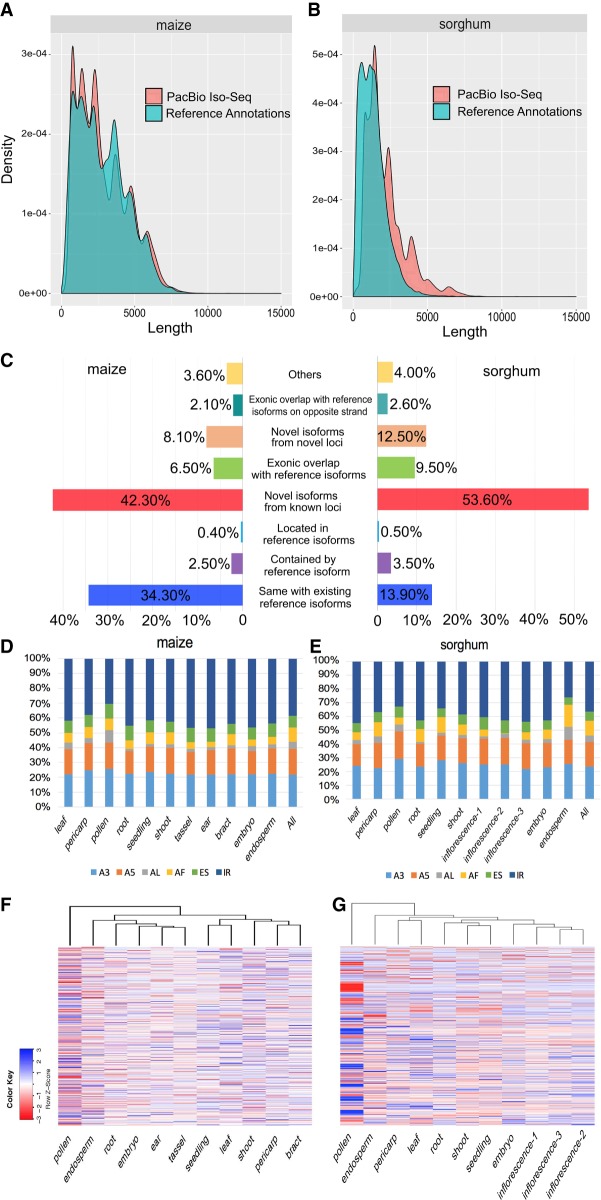
Characterization of maize and sorghum isoforms from PacBio Iso-Seq. (*A*) Comparison of transcript length distributions between maize reference annotations and Iso-Seq isoforms. (*B*) Comparison of transcripts length distributions between sorghum reference annotations and Iso-Seq isoforms. (*C*) Classification of Iso-Seq isoforms of maize and sorghum. (*D*) Distribution of splicing patterns across tissues in maize. (*E*) Distribution of splicing patterns across tissues in sorghum. (*F*) Heatmap of PSI values across tissues in maize. (*G*) Heatmap of PSI values across tissues in sorghum. (ES) Exon skipping; (A5) alternative 5′ splice-site; (A3) alternative 3′ splice-site; (IR) intron retention; (AF) alternative first exon; (AL) alternative last exon.

Comparison of transcript length distributions revealed that a large number of novel long transcripts captured in sorghum compared to maize since the latter was annotated using previously sequenced PacBio transcripts (Fig. 1A,B). This constituted nearly fourfold enrichment relative to the annotation, comparable to a similar discovery in maize reported by our group (Wang et al. 2016).

We classified the isoforms into eight groups (Fig. 1C): (1) novel transcripts from novel loci (i.e., absent from previous annotations, V4 or Sbi1.4); (2) novel isoforms that share at least one splice site with annotated genes/isoforms but differ at other splice sites; (3) isoforms that share the same intron chain and splice sites with existing gene models; (4) isoforms with exonic overlap with existing gene models but without shared splice sites; (5) PacBio isoforms located in introns of annotated isoforms; (6) isoforms with exonic overlap with an annotated locus on the opposite strand; (7) isoforms partially matching annotated transcripts, i.e., parts of transcripts sharing splice sites at matched regions but shorter than annotated sequences; and (8) others not belonging to any of the above categories.

### Tissue-specific isoforms and AS activity

We also studied isoform specificity and commonality among different tissues at different developmental stages. We found that 1659 isoforms in maize and 1069 in sorghum were shared by all 11 tissues. In maize, pollen tissue had the highest proportion of tissue-specific isoforms (27.2%), and root had the smallest (14.5%). In sorghum, inflorescence-1 tissue had the highest proportion of specific isoforms (35.8%), slightly more than in pollen (34.1%), and (as in maize) root had the smallest (20.8%). We validated the isoforms using Illumina RNA-seq, revealing that most of the Iso-Seq transcripts are very well supported by short-read sequencing, with 95% and 92% of the splice sites supported in sorghum and maize, respectively. Pollen tissue has the least well-supported splicing sites in both species (Supplemental Fig S1).

AS plays important roles in mRNA processing. We found that 18,741 (45%) and 13,327 (38.5%) of expressed genes were alternatively spliced in maize and sorghum, respectively. To ascertain the relative importance of different types of AS in each tissue, we investigated AS events using the SUPPA pipeline (Alamancos et al. 2015). Overall, intron retention (IR) was the predominant splicing pattern in most tissues, with alternative 3′ splice sites ranking second and alternative last (AL) exon ranking last, but the proportion of different splicing events varied among tissues (Fig. 1D,E). To examine the variation of splicing events throughout development, we used RNA-seq data to calculate a “percent splicing index” (PSI) value per tissue for each AS event. PSI measures the supporting isoform(s) as a percentage of total isoforms. Differences in PSI among tissues reveal the diversity of splicing activity (Cheng et al. 2017). The results revealed that many splicing events were specific to particular tissue(s) and thus may be regulated in a tissue-specific manner. Notably in this regard, pollen had the highest splicing activity among all tissues, as reflected by its high *Z*-score (Fig. 1F,G), as well as the highest splicing activity across all splicing patterns in both species (Supplemental Figs. S2, S3).

### AS events coupled with nonsense-mediated mRNA decay

**Figure 2.**
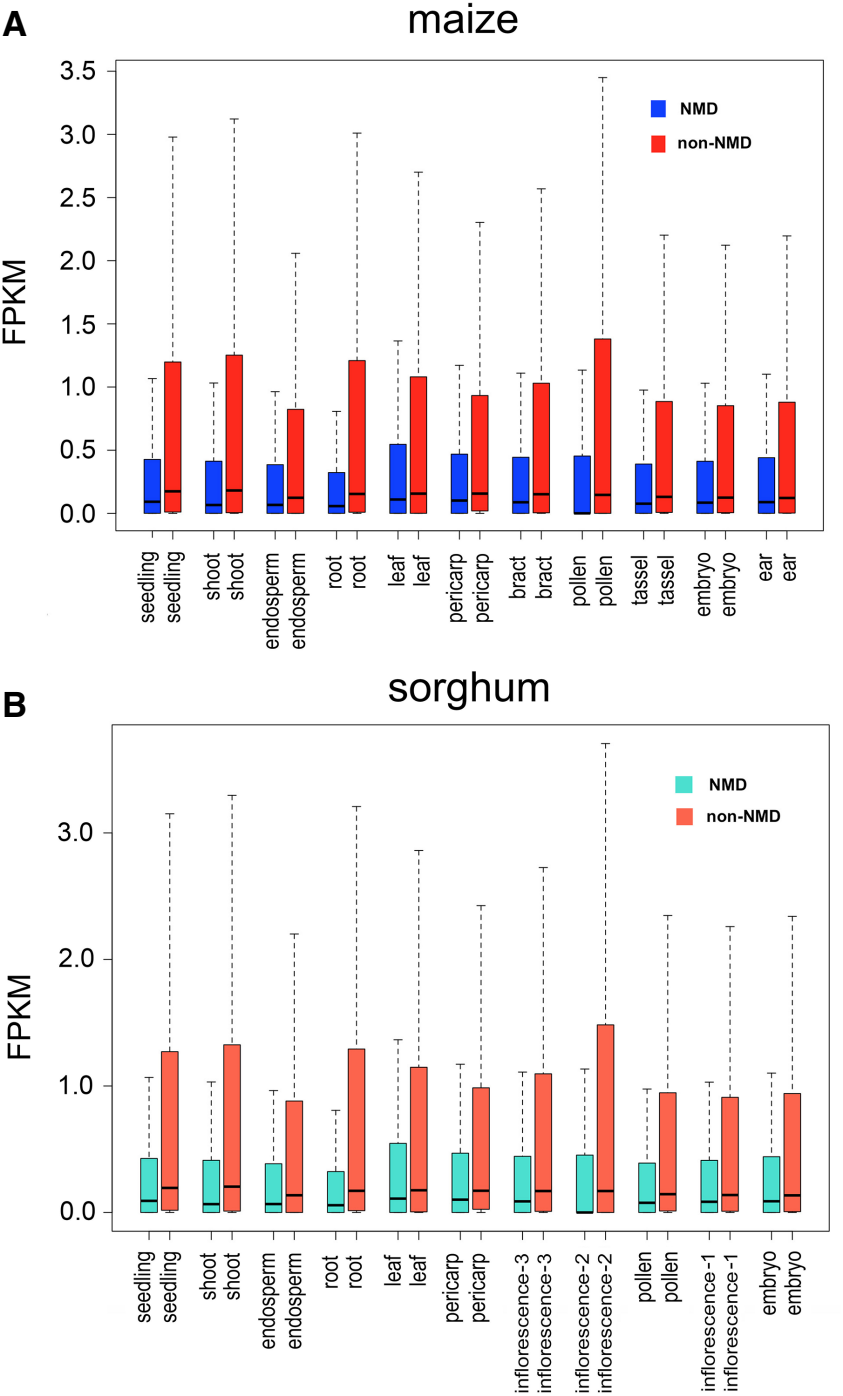
Comparison of expression levels between NMD and non-NMD isoforms. Comparison of expression level between NMD and non-NMD isoforms among tissues in maize (*A*) and sorghum (*B*).

Nonsense-mediated decay (NMD) is a cellular process that targets mRNAs carrying a premature termination codon (PTC) for degradation. Through single-molecule sequencing, we identified a large number of isoforms, which mostly have altered open reading frames with early stop codons, as candidates for NMD: 55,080 (40.3%) out of 136,745 maize isoforms and 34,322 (36%) out of 95,380 sorghum isoforms. Overall, non-NMD isoforms were more highly expressed than NMD isoforms in both species (Fig. 2A,B). The proportion of NMD candidates differed among isoforms with various splicing patterns and was highest in IR isoforms in both species (Supplemental Fig S4A,B). The expression levels of NMD isoforms with different splicing patterns exhibited no significant differences among tissues.

### Conserved AS between maize and sorghum

To elucidate the pattern of conserved isoforms between maize and sorghum, we first identified 21,943 sorghum isoforms that exhibited conservation with maize isoforms. Maize and sorghum exhibited similar splicing patterns, with IR predominant and AL least common (Supplemental Fig. S5A). Among the conserved isoforms, 2068 IR events were conserved, indicating that these events are likely to be biologically functional. To determine how many conserved isoforms are NMD candidates, we investigated the conserved isoforms of each splicing pattern in the two species. Among all the conserved splicing patterns, IR isoforms were more likely to be NMD candidates in both species; however, isoforms with conserved ES patterns had the least proportion compared with other patterns, especially in sorghum (Supplemental Fig. S4C,D).

Orthologous genes had more isoforms in maize than in sorghum (Supplemental Fig. S5B). In maize, singletons and duplicated genes had similar numbers of isoforms (Supplemental Fig. S6A) and similar expression levels in each tissue, with overall slightly higher expression of duplicated genes in most tissues (Supplemental Fig. S6B). Of the two maize subgenomes, the dominant subgenome A experienced fewer deletions than subgenome B (Schnable et al. 2009; Pophaly and Tellier 2015). Comparison of the subgenomes A and B revealed no bias in isoform generation (Supplemental Fig. S7A); however, genes in pollen were more highly expressed in subgenome B and genes in endosperm were more highly expressed in subgenome A (Supplemental Fig. S7B).

### Gene Ontology analysis

Gene Ontology (GO) analysis revealed that various tissues exhibited enrichment in genes associated with different GO terms, potentially related to the functions of a given tissue during plant development. For example, the GO term “nutrient reservoir” was enriched in endosperm of both maize and sorghum. We also detected differences between maize and sorghum for the same tissues. For example, the biological process term “response to stress” was enriched in sorghum root but not in maize root, indicating a functional difference between the species (Supplemental Figs. S8, S9).

### Transcription factor isoforms produce functional variants in both maize and sorghum

Transcription factors play a critical role in plant development. Maize has 57 transcription factor families, and sorghum has 43 (Yilmaz et al. 2009). By using our single-molecule sequencing data, we identified novel isoforms from 53 families in maize and all 43 families of sorghum (Supplemental Fig. S10A). In both species, some families had particularly high prevalence of isoforms; e.g., in the MYBR family, we found 129 novel isoforms in sorghum and 179 in maize (Supplemental Fig. S10B). The predominance of splicing patterns of transcription factors was similar to overall splicing patterns between species: IR ranked highest, and AL lowest (Supplemental Fig. S11). Similarly, expression profiles of transcription factors showed that different TFs have different preferences for expression in various tissues and that pollen has the highest expression of TFs among tissues (Supplemental Fig. S12A,B). GO analysis also revealed differences in functional classes between maize and sorghum (Supplemental Fig. S12C).

### Analysis of the complexity of transcriptome diversity in maize and sorghum

**Figure 3.**
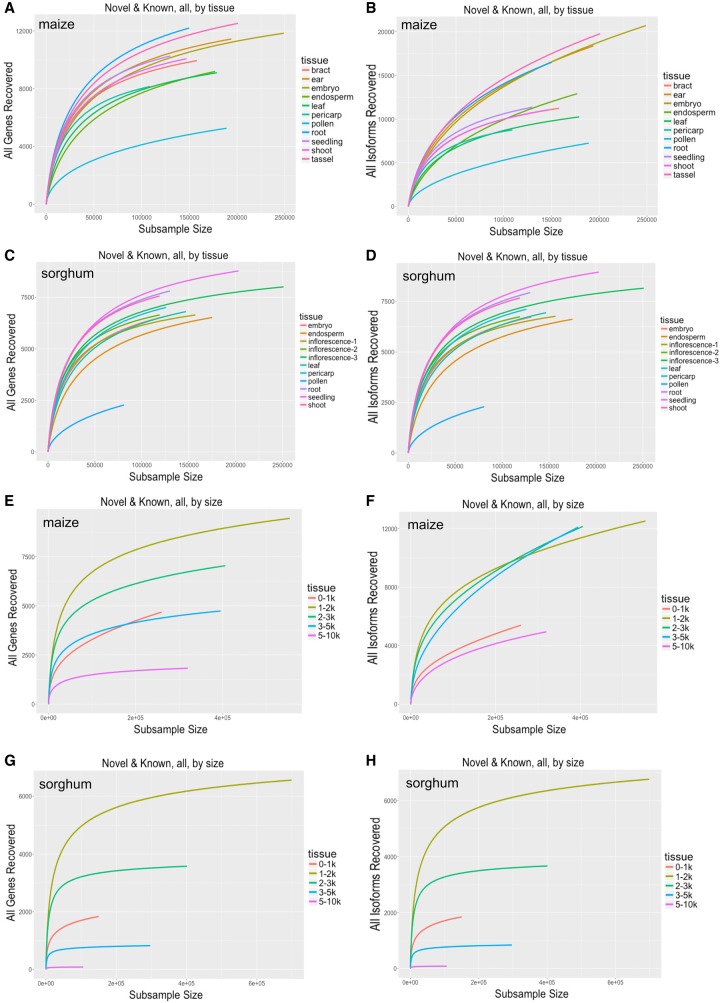
Rarefaction analysis of covered genes/isoforms in maize and sorghum. Rarefaction analysis of the following: (*A*) covered genes in maize tissues, (*B*) covered isoforms in maize tissues, (*C*) covered genes in sorghum tissues, (*D*) covered isoforms in sorghum tissues, (*E*) covered genes in maize across size-fractionated libraries, (*F*) covered isoforms in maize across size-fractionated libraries, (*G*) covered genes in sorghum across size-fractionated libraries, and (*H*) covered isoforms in sorghum across size-fractionated libraries.

To determine the complexity of various tissues within each species, and to identify differences between matched tissues between the two species, we performed rarefaction analysis on all tissues in each size-fractionated library. The sequencing depth achieved using 135 multiplexed SMRT (single-molecule real-time sequencing) cells reached near-saturation of gene discovery within all size ranges. Saturation was easier to reach in tissues of sorghum due to its lower genome complexity and gene number (Fig. 3A–D). In contrast, due to the complexity of the maize genome, it was harder to achieve saturation of sequenced isoforms (Fig. 3E–H).

To further investigate the relationship between sequencing depth and transcript discovery, we performed similar analyses using reads from individual tissues or different size-fractionated libraries, pooled across tissues. In both maize and sorghum, seedling-related tissues exhibited the greatest transcriptome diversity at shorter transcript ranges, whereas adult tissues exhibited the greatest diversity at longer transcript ranges. Discovery of known and novel RefGen_v4 transcript isoforms reached saturation much sooner (i.e., at lower subsample sizes) in pollen in both species (Supplemental Figs. S13–S16). Saturation was also reached sooner in reads from larger-insert libraries, with a clear trend toward decreasing transcript diversity with increasing insert length, except that the 1- to 2-kb library was more diverse than the 0- to 1-kb library. In addition, the transcriptome complexity of tissues varied among size fractions. Similar results were obtained using novel and known transcript isoforms as outcome measures.

### Alternative polyadenylation in maize and sorghum tissues

**Figure 4.**
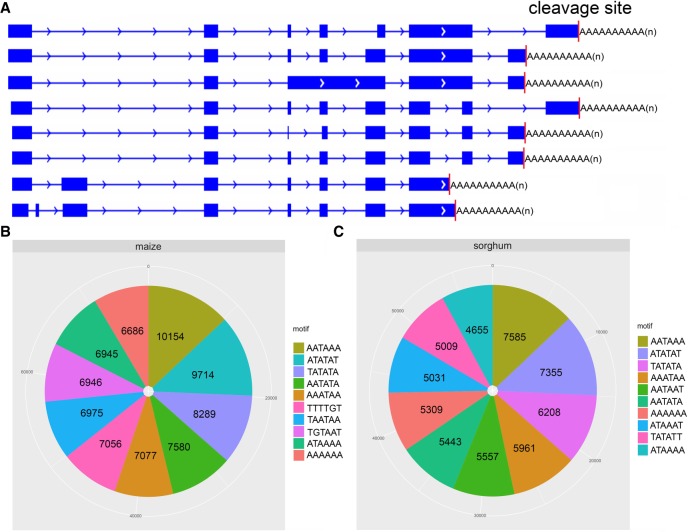
Characterization of alternative polyadenylation (APA) in maize and sorghum. (*A*) Diversity of cleavage sites among different isoforms of sorghum gene *Sb05g022140* in region Chr 5: 53,742,544–53,755,286. Top 10 APA motifs are shown for maize (*B*) and sorghum (*C*).

Alternative polyadenylation (APA) is a common regulatory mechanism of transcriptome complexity. Although alternative poly(A) signals are common in maize and sorghum (Fig. 4A), they remain largely uncharacterized. To investigate the role of poly(A) motifs in the maize and sorghum transcriptomes, we first counted the poly(A) cleavage positions. We found that many genes have diverse poly(A) cleavage sites (CSs) (Supplemental Fig. S17A,B) and that the CSs of poly(A) vary among tissues (Supplemental Fig. S18A,B). Next, to determine which motifs are in charge of poly(A) adenylation, we used SignalSleuth2 (Zhao et al. 2014) to scan the near upstream element (NUE) regions of full-length transcripts expressed in each tissue. The results revealed that the AATAAA motif is predominant and that the top three motifs were the same in both species; however, the relative ranks of some motifs differed; e.g., AATATA ranked fourth in maize but sixth in sorghum (Fig. 4B,C). Comparison of APA motifs in orthologous genes between species (Supplemental Fig. S19) and genes among tissues within each species (Supplemental Figs. S20, S21) revealed that various tissues exhibit differences in poly(A) signal generation and that similar tissues in different species can use different mechanisms to generate poly(A) cleavage signals. Considering polyadenylation proteins are key regulator of polyadenylation machinery, we then investigated the gene expression of cleavage and polyadenylation factors in both maize and sorghum. We found that these factors have diverse expression pattern among different tissues in both maize and sorghum (Supplemental Fig. S22A,B). To further characterize the tissue specificity of polyadenylation, we clustered full-length transcripts ending within five nucleotides of each other across all 11 tissues in both species. The number of tissue-specific poly(A) sites and genes containing such sites differed among tissues in a species-specific manner (Supplemental Fig. S23A,B). Together, these phenomena contribute to the transcript diversity among tissues and species.

### Identification and comparative analysis of lncRNAs

**Figure 5.**
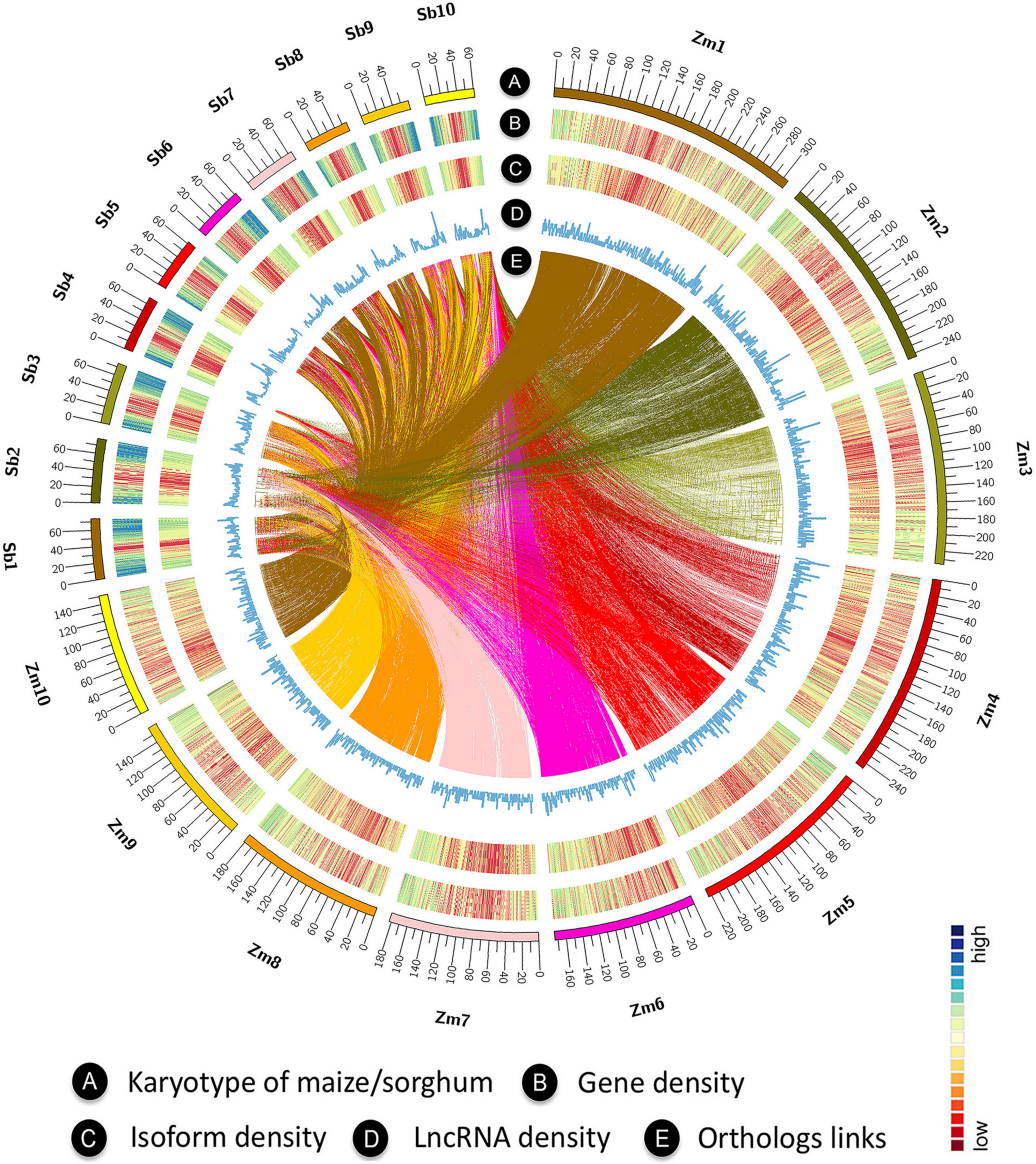
Circos (Krzywinski et al. 2009) visualization of comparative features between maize and sorghum genomes. (*A*) Karyotype of maize and sorghum. (*B*) Genome-wide distribution of gene density in maize and sorghum. (*C*) Genome-wide distribution of isoform density in maize and sorghum. (*D*) Genome-wide distribution of lncRNA density in maize and sorghum. (*E*) Ortholog links between maize and sorghum.

LncRNAs play a variety of biological roles in organisms. The larger number of tissues used in this study enabled the discovery of more novel lncRNAs in both species. To this end, we used a previously described strategy (Wang et al. 2016) to build a classification model using PLEK (Li et al. 2014a), trained on high-confidence sets of known noncoding RNA genes (Li et al. 2014b; Abdel-Ghany et al. 2016; Wang et al. 2016). Application of this model to sorghum reads, followed by removal of potentially protein-coding sequences, yielded 1706 novel high-confidence lncRNAs with median lengths of 1241 bp (range, 305–7563 bp) (Supplemental Fig. S27A), longer than those previously identified by single-molecule sequencing (median length, 880 bp) (Abdel-Ghany et al. 2016). By using the same strategy, we identified 39 high-confidence lncRNAs in maize, in addition to those discovered in the previous study. Overall, sorghum lncRNAs (median length, 1119 bp) were longer than those of maize (median length, 535 bp) (Supplemental Fig. S27B). Maize and sorghum lncRNAs were distributed similarly along chromosomes, consistent with the genes/isoforms distribution (Fig. 5A–D). Only five lncRNAs from sorghum exhibited good conservation with maize, based on criteria of ≥70% coverage and ≥80% identity (Supplemental Table S3).

### Gene expression is more similar between comparable tissues of both species than within tissues of each species

**Figure 6.**
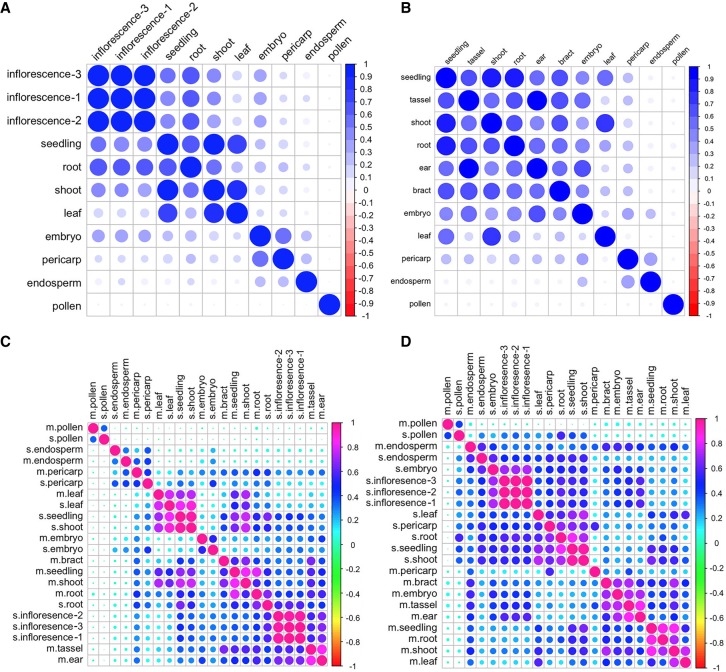
Correlation matrix of genes among tissues, within and between species. Correlation matrices of the following: (*A*) gene expression among tissues in sorghum, (*B*) gene expression among tissues in maize, (*C*) gene expression among tissues between maize and sorghum, and (*D*) transcription factor expression among tissues between maize and sorghum.

To explore the similarities and differences in gene expression between maize and sorghum, we performed correlation analysis based on gene expression levels in each species. The results revealed clustering of developmentally closely related tissues in sorghum (Fig. 6A) but not in maize (Fig. 6B). Based on one-to-one orthologs (Fig. 5E), we generated a correlation matrix between maize and sorghum for all investigated tissues. Comparable tissues between species were more likely to be clustered than tissues within a species (Fig. 6C). This effect was also observed for single-copy genes in maize subgenomes A and B but with slight differences for duplicated genes with copies in both subgenomes (Supplemental Fig. S24A–C). Given that transcription factors play important roles in multiple aspects of plant development, we clustered the maize and sorghum transcription factors using one-to-one orthologs. We found that some tissues clustered together, e.g., pollen and endosperm between maize and sorghum, whereas most others did not (Fig. 6D). We also observed differences between the maize subgenomes A and B, which were even more prominent for duplicated genes existing in both subgenomes (Supplemental Fig. S25A–C). Therefore, transcription factor expression could play a role in the developmental divergence of similar tissues in maize and sorghum, and this effect could be buffered by expression of other non-TF genes. In addition, housekeeping genes had significantly more isoforms than tissue-specific genes in both species (Supplemental Fig. S26A,B). Tissue-specific genes had the highest numbers of isoforms in pericarp of maize and in embryo of sorghum (Supplemental Fig. S26C,D).

### Evolutionary age of maize and sorghum transcriptome

**Figure 7.**
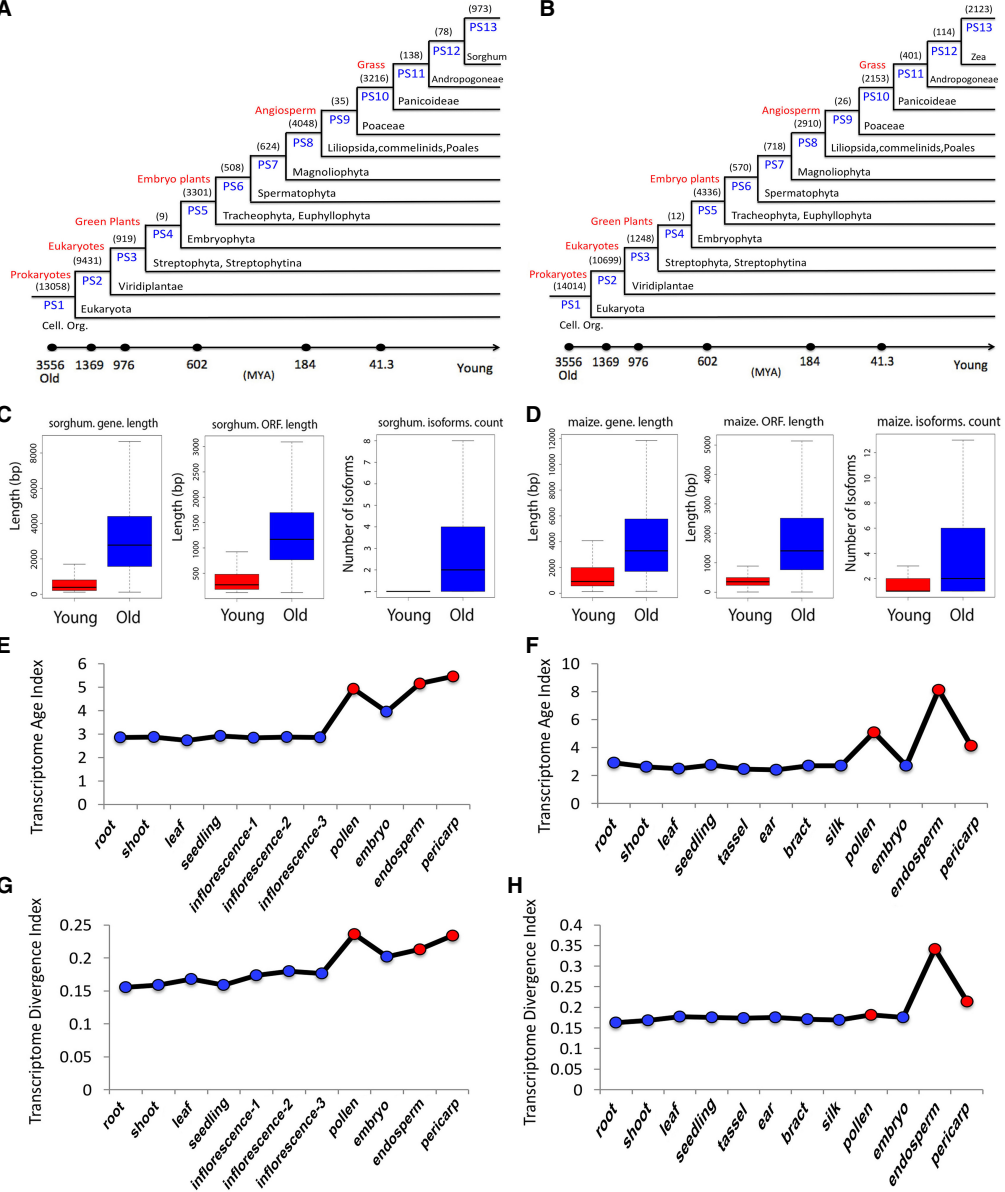
Phylogratigraphic profile of genes in maize and sorghum and comparison of features between young and old genes. Phylostratigraphic profile of genes in sorghum (*A*) and maize (*B*). Comparison of gene/ORF length and number of isoforms between old and young genes in sorghum (*C*) and maize (*D*). (*E*,*F*) TAI of each tissue in sorghum (*E*) and maize (*F*). (*G*,*H*) TDI of each tissue in sorghum (*G*) and maize (*H*).

To investigate changes in gene expression patterns over the course of evolution, we calculated the transcriptome age of each tissue in maize and sorghum using a previously reported approach (Domazet-Lošo and Tautz 2010; Quint et al. 2012; Drost et al. 2015, 2016, 2018). For this purpose, we first built phylostratigraphic profiles of genes for each species (Fig. 7A,B). By using this phylogenetic hierarchy, we assigned each gene a phylostratum (PS) value. For comparison and simplicity, we defined all genes from PS1 to PS3 as “old,” and those from PS11 to PS13 as “young.” Thus, in maize, 59.2% of the protein-coding genes are old and 3% are young, whereas in sorghum, 66% are old and 6.7% are young. Overall, maize has more young genes (2123 species-specific orphan genes [SSOGs]), 5.4%; 515 taxon-specific orphan genes [TSOGs], 1.3%) than sorghum (973 SSOGs, 2.5%; 216 TSOGs, 0.5%). In both species, old genes and their ORFs are longer and have more isoforms in comparison to young genes (Fig. 7C,D).

In addition to the Iso-Seq transcripts, we generated a series of RNA-seq transcriptome data sets for maize and sorghum across different developmental stages. The transcriptome age index (TAI), which quantifies the mean evolutionary age of a transcriptome, and transcriptome divergence index (TDI), which represents the mean sequence divergence of a transcriptome, were calculated using the PS value and expression level of each gene across tissues as previously reported (Quint et al. 2012; Drost et al. 2015, 2016, 2018). In both maize and sorghum, reproductive tissues had much higher TAI and TDI values than vegetative tissues. Overall, tissues with higher TAI values also had higher TDI values, but each tissue exhibited slight differences between maize and sorghum (Fig. 7E–H). TAI values ranged from 2.56 (leaf) to 5.49 (pollen) in sorghum (Fig. 7E), from 2.78 (leaf) to 6.23 (endosperm) (Fig. 7F) in maize. The lowest TAI values were observed in the leaf transcriptomes of both maize and sorghum, whereas the highest were observed in different tissues in each species. However, the top three TAI values for both species were from the same tissues (pollen, endosperm, and pericarp), suggesting that these tissues have the evolutionarily youngest transcriptomes. These differences also correspond well with the observation that in early developmental stages, the architectures of maize and sorghum are similar, whereas in later stages the two species have very divergent morphologies, with the male and female reproductive tissues on separate inflorescences. Gene expression patterns across tissues in the same PS ranks revealed that old genes were more highly expressed than younger genes. Furthermore, the expression patterns of old genes were similar across tissues, as was the case for younger genes. Expression of PS4 genes was much higher in root than in any other tissue in both species (Supplemental Figs. S28, S29).

To determine whether the expression patterns of genes in different PS classes vary within the same tissue, we examined the expression of genes in 13 PS ranks in each tissue. Again, old genes were more highly expressed than young genes, irrespective of tissue. In maize, genes of class PS4 were very weakly expressed in all tissues except for root, whereas in sorghum, PS4 genes were expressed in pollen, root, and seedling. Old genes had almost twice as many isoforms as young genes, and old genes had more isoforms in both vegetative and reproductive tissues (Supplemental Figs. S30, S31). Moreover, more highly expressed genes did not necessarily have more isoforms in both species, irrespective of tissue type (Supplemental Figs. S32, S33).

## Discussion

This study represents the first large-scale comparative analysis of the transcriptome by single-molecule long-read sequencing in matched tissues in maize and sorghum. Previous studies revealed the complexity of the maize and sorghum transcriptomes, using either short-read sequencing or single-molecule sequencing (Abdel-Ghany et al. 2016; Wang et al. 2016; Mei et al. 2017). Our results indicate that isoform characterization in both maize and sorghum remains far from complete and suggest that isoform diversification between similar tissues contributes to differentiation of plant architecture. A major challenge for the future will be to determine which differences in expression levels of splice variants, as well as which particular isoforms, are associated with species-specific functional and phenotypic attributes. This challenge also pertains to analyses of recently reported differences on gene structure variations and other levels of gene regulation.

To what degree are evolutionary changes in AS or gene expression required to establish major differences in morphological and phenotypic characteristics? Changes with small effects at multiple loci probably underlie many species-specific differences, but individual differences in the expression of genes involved in developmental processes can also drive the evolution of major morphological diversification (Calarco et al. 2007). The differences we detected in gene expression and splicing levels in matched tissues could have a significant impact on the morphological and other phenotypic differences between maize and sorghum, which presumably reflect the underlying biology of the two species. For example, maize and sorghum have very different metabolisms and physiologies: Maize is a major summer crop, has high water requirements, is highly productive if water and nutrients are not limiting, and is very sensitive to water stress (Rhoads and Bennet 1990). In contrast, sorghum, which is grown as a summer crop in agronomic conditions similar to those of maize, is drought tolerant (Krieg and Lascano 1990) largely due to its prolific root system, phenology, and osmotic adjustment to water stress (Girma and Krieg 1992; Singh and Singh 1995). Our results provide the basis for future investigations aimed at elucidating the functional and phenotypic consequences of AS differences between maize and sorghum, as well as other evolutionarily closely related species.

In previous studies, we demonstrated that large-scale full-length cDNA sequencing is a powerful tool for characterization of AS, gene discovery, and genome annotation (Hirsch et al. 2014; Tello-Ruiz et al. 2016; Wang et al. 2016). This study was designed to compare full-length transcriptome and expression profiles between maize and sorghum with a focus on evolutionary developmental biology. Most of the previous studies focused on conservation of gene expression among species, and very little work has been done at the level of AS, especially in plants. Thus, our sequencing strategy complements existing resources and provides advantages for discovery of novel or previously unrecognized protein-coding genes and transcript isoforms. Moreover, our results demonstrate that full-length transcriptome data have enormous potential to improve the current maize and sorghum annotations and could contribute to our understanding of AS in both species.

Our findings also shed light on the importance of APA in transcriptome diversification between maize and sorghum. Recent high-throughput studies revealed that APA enhances transcriptome complexity by generating transcript isoforms that differ in the coding or 3′ untranslated regions, thereby regulating gene expression via multiple mechanisms in both plants and animals (Shen et al. 2011; Wu et al. 2011; Elkon et al. 2013; Abdel-Ghany et al. 2016). Differential polyadenylation of mRNAs plays an important regulatory role in plant development, especially flowering (Simpson et al. 2003; Liu et al. 2010). Although identification of the 3′ ends of the transcripts from a gene is essential for a comprehensive analysis of differential polyadenylation, it is possible to precisely identify APA sites using short-read data. In *Arabidopsis*, specialized poly(A) tag sequences and direct RNA sequencing have been used to identify transcript isoform differences due to APA (Wu et al. 2011). The maize and sorghum transcriptomes have been extensively studied using high-throughput sequencing methods (Sekhon et al. 2011; Gelli et al. 2014; Shakoor et al. 2014; Stelpflug et al. 2016); to date, however, only a few reports have compiled 3′-end information on a genome-wide scale. In this work, we generated comprehensive and high-resolution maps of genome-wide poly(A) sites, allowing systematic characterization of the role of APA in 11 agronomically important tissues from each of the two species examined. In addition, we generated comprehensive APA profiles of each tissue at different developmental stages in both species. Consistent with discoveries in rice (Shen et al. 2008), the AATAAA motif in the NUE region is predominant in both maize and sorghum. However, we detected differences in APA motifs among tissues, especially in pollen, where ATATAT predominates, indicating that different tissues exploit different mechanisms for mRNA cleavage. On the other hand, in contrast to inflorescence tissues in sorghum, ear and tassel tissue in maize are separated during development; however, the ATATAT motif predominates in all of these tissues, indicating that a similar mRNA processing signal operates during development of reproductive tissues in both maize and sorghum. This feature might not be closely related to inflorescence divergence in terms of the evolution of sex determination in these two species, even though specific isoforms might play important roles in this phenomenon, as reported in *Drosophila* (Li et al. 2004).

Finally, in a higher-level analysis of the evolution of gene expression, we calculated the transcriptome age of each tissue in both species and found that the transcriptomes of reproductive tissues are much younger than those of vegetative tissues. In addition, we identified the tissues with the youngest transcriptome ages. A previous study (Kaessmann 2010) revealed that in animals, the male reproductive organ acts as a major source of new genes. We obtained evidence of pollen-biased expression of young genes in monocot plants, suggesting that a common mechanism drives the emergence of young genes in male reproductive tissues, as also hypothesized in rice and *Arabidopsis* (Wu et al. 2014; Cui et al. 2015). However, we also observed differences among species. For example, in maize unlike in sorghum, endosperm's transcriptome is younger than in other tissues, including pollen, suggesting that different mechanisms drive gene evolution in each species.

Overall, our study reveals extensive divergence in the expression of both protein-coding and noncoding genes, as well as differential AS, which together are likely to explain the extensive morphological and functional differences between maize and sorghum.

## Methods

### Plant materials

Maize inbred line B73 and sorghum BTx623 were grown at CSHL Uplands Farm. For maize, root, shoot, leaf, and seedling were collected at 14-d-old stage; ears from stage v8; tassels from stage v7; pollen from stage r1; embryo, endosperm, and pericarp from seeds 20 d after pollination; silk from the R1 stage; and bract from the innermost husk. For sorghum tissues, root, shoot, leaf, and seedling were collected 14 d after germination; embryo, endosperm, and pericarp 20 d after pollination; and pollen at the 9- to 10-wk stage. Inflorescences were collected at the following sizes: 1–5 mm (inflorescence-1), 5–10 mm (inflorescence-2), and 1–2 cm (inflorescence-3). All tissues were immediately frozen in liquid N_2_. For each tissue, at least 10 plants were pooled in each of three biological replicates.

### Standard methods

The standard methods such as RNA preparation, Illumina RNA-seq library construction, PacBio library construction and single-molecule sequencing, Illumina short-reads data analysis, mapping of PacBio data, lncRNA identification from PacBio sequences, and functional annotation of PacBio isoforms have been discussed previously (Wang et al. 2016) with detailed modifications in the Supplemental Methods section.

### Identification of singletons and duplicates

Singletons and duplicates were determined using homology between the maize and sorghum genome based on the Ensembl Compara gene tree pipeline (Tello-Ruiz et al. 2016). We used the sorghum genes as the ancient gene set. If a sorghum gene only had one maize ortholog, the maize gene was considered as singleton. If two maize genes were orthologous to one sorghum gene, the maize genes were considered to be a pair of duplicated genes.

### APA analysis

The sequences 50 nt upstream of the CS in the 3′ UTR of each transcript were extracted, and SignalSleuth2 (Zhao et al. 2014) was used to scan the poly(A) trigger motif from 1–40 nt upstream CS region to identify the NUE motif. The top 10 motifs from each tissue were used for comparison between species and among tissues. To measure the tissue specificity of polyadenylation, we clustered full-length transcripts ending within 5 nt of each other across all 11 tissues in both species. The number of different tissues with transcripts ending in each of these poly(A) site regions were tallied and grouped by gene locus.

### Prediction of NMD candidates

To determine whether an AS event generated an isoform that contains premature stop codons and could be degraded by NMD, we first predicted the longest ORF of each isoform by EMBOSS (Rice et al. 2000) and then calculated the distance between the stop codon and the last exon junction for each isoform. If one isoform had a distance >50 nt whereas the other had a distance <50 nt, then the AS event was regarded as producing an NMD candidate (Wang and Brendel 2006).

### *K*_a_/*K*_s_ ratios

For each organism, we randomly picked at least one homologous strain to find pairs of orthologous proteins with an *e*-value ≤10^−5^ based on BLASTP searches and then selected the orthologous protein with the highest BLASTP score for further analysis. Pairs of protein sequences were aligned by Clustal W 2.0 (Larkin et al. 2007) with default options, and protein sequence alignments were converted to the corresponding codon (i.e., nucleotide) alignments using Pal2Nal (Suyama et al. 2006). *K*_a_/*K*_s_ value was calculated using KaKs_Calculator1.2 (Wang et al. 2010) according to the Nei–Gojobori method.

### TAI and TDI

The TAI and the TDI are weighted means of evolutionary age and sequence divergence, respectively, and are defined as in Domazet-Lošo and Tautz (2010), Quint et al. (2012), and Drost et al. (2015, 2016, 2018). TAI_*s*_ of developmental stage *s* (*s* = each tissue) is the weighted mean of the evolutionary age (phylostratum) *ps*_*i*_ of gene *i* weighted by the expression level *e*_*is*_ of gene *i* at developmental stage *s*:

$${\text{TAI}}_{s}=\frac{\sum_{i=1}^{n}\;ps_{i}e_{is}}{\sum_{i=1}^{n}e_{is}},$$

where *n* is the total number of genes analyzed. Low PS values correspond to evolutionarily old genes, so low TAI values correspond to evolutionarily old transcriptomes. Likewise, high PS values correspond to evolutionarily young genes, so high TAI values correspond to evolutionarily young transcriptomes. By analogy, the transcriptome divergence index TDI_*s*_ of developmental stage *s* simply was calculated by replacing *ps*_*i*_ in the above equation by the *K*_a_/*K*_s_ ratio of gene *i*:

$${\text{TDI}}_{s}=\frac{\sum_{i=1}^{n}\left(\frac{K_{ai}}{K_{si}}\right)e_{is}}{\sum_{i=1}^{n}e_{is}}.$$

Hence, low or high *K*_a_/*K*_s_ ratios correspond to conserved or divergent genes, respectively; so low or high TDI values correspond to conserved or divergent transcriptomes, respectively. The same procedure was repeated for the second independent data set covering different tissues of maize.

## Data access

The data generated in this study, including PacBio Iso-Seq reads and Illumina short reads, have been submitted to ArrayExpress (https://www.ebi.ac.uk/arrayexpress/) under accession numbers E-MTAB-5957, E-MTAB-5915, and E-MTAB-5956.

## Competing interest statement

E.T. is a full-time employee of Pacific Biosciences. W.R.M. has participated in Illumina sponsored meetings over the past four years and received travel reimbursement and an honorarium for presenting at these events. Illumina had no role in decisions relating to the study/work to be published, data collection and analysis of data and the decision to publish. W.R.M. has participated in Pacific Biosciences sponsored meetings over the past three years and received travel reimbursement for presenting at these events. W.R.M. is a founder and shared holder of Orion Genomics, which focuses on plant genomics and cancer genetics. W.R.M. is a SAB member for RainDance Technologies, Inc.

## Supplementary Material

Supplemental Material
