# Supplementary material for: A comparative transcriptional landscape of maize and sorghum obtained by single-molecule sequencing
Source: Genome Res. 2018 Jun;28(6):921–32. doi: 10.1101/gr.227462.117 (PMC5991521; doi:10.1101/gr.227462.117)
Supplement: Supplemental Material [file supp_28_6_921__index.html]

A comparative transcriptional landscape of maize and sorghum obtained by single-molecule sequencing — Supplemental Material 

# A comparative transcriptional landscape of maize and sorghum obtained by single-molecule sequencing

## Supplemental Material

- Supplemental\_Fig\_S1.pdf
- Supplemental\_Fig\_S2.pdf
- Supplemental\_Fig\_S3.pdf
- Supplemental\_Fig\_S4.pdf
- Supplemental\_Fig\_S5.pdf
- Supplemental\_Fig\_S6.pdf
- Supplemental\_Fig\_S7.pdf
- Supplemental\_Fig\_S8.pdf
- Supplemental\_Fig\_S9.pdf
- Supplemental\_Fig\_S10.pdf
- Supplemental\_Fig\_S11.pdf
- Supplemental\_Fig\_S12.pdf
- Supplemental\_Fig\_S13.pdf
- Supplemental\_Fig\_S14.pdf
- Supplemental\_Fig\_S15.pdf
- Supplemental\_Fig\_S16.pdf
- Supplemental\_Fig\_S17.pdf
- Supplemental\_Fig\_S18.pdf
- Supplemental\_Fig\_S19.pdf
- Supplemental\_Fig\_S20.pdf
- Supplemental\_Fig\_S21.pdf
- Supplemental\_Fig\_S22.pdf
- Supplemental\_Fig\_S23.pdf
- Supplemental\_Fig\_S24.pdf
- Supplemental\_Fig\_S25.pdf
- Supplemental\_Fig\_S26.pdf
- Supplemental\_Fig\_S27.pdf
- Supplemental\_Fig\_S28.pdf
- Supplemental\_Fig\_S29.pdf
- Supplemental\_Fig\_S30.pdf
- Supplemental\_Fig\_S31.pdf
- Supplemental\_Fig\_S32.pdf
- Supplemental\_Fig\_S33.pdf
- Supplemental\_Table\_S1.pdf
- Supplemental\_Table\_S2.pdf
- Supplemental\_Table\_S3.pdf
- Supplemental\_Table\_S4.pdf
- Supplemental\_Methods.pdf
