# Supplementary material for: A comparative transcriptional landscape of maize and sorghum obtained by single-molecule sequencing
Source: Genome Res. 2018 Jun;28(6):921–32. doi: 10.1101/gr.227462.117 (PMC5991521; doi:10.1101/gr.227462.117)
Supplement: Supplemental Material [file supp_gr.227462.117_Supplemental_Fig_S1.pdf]

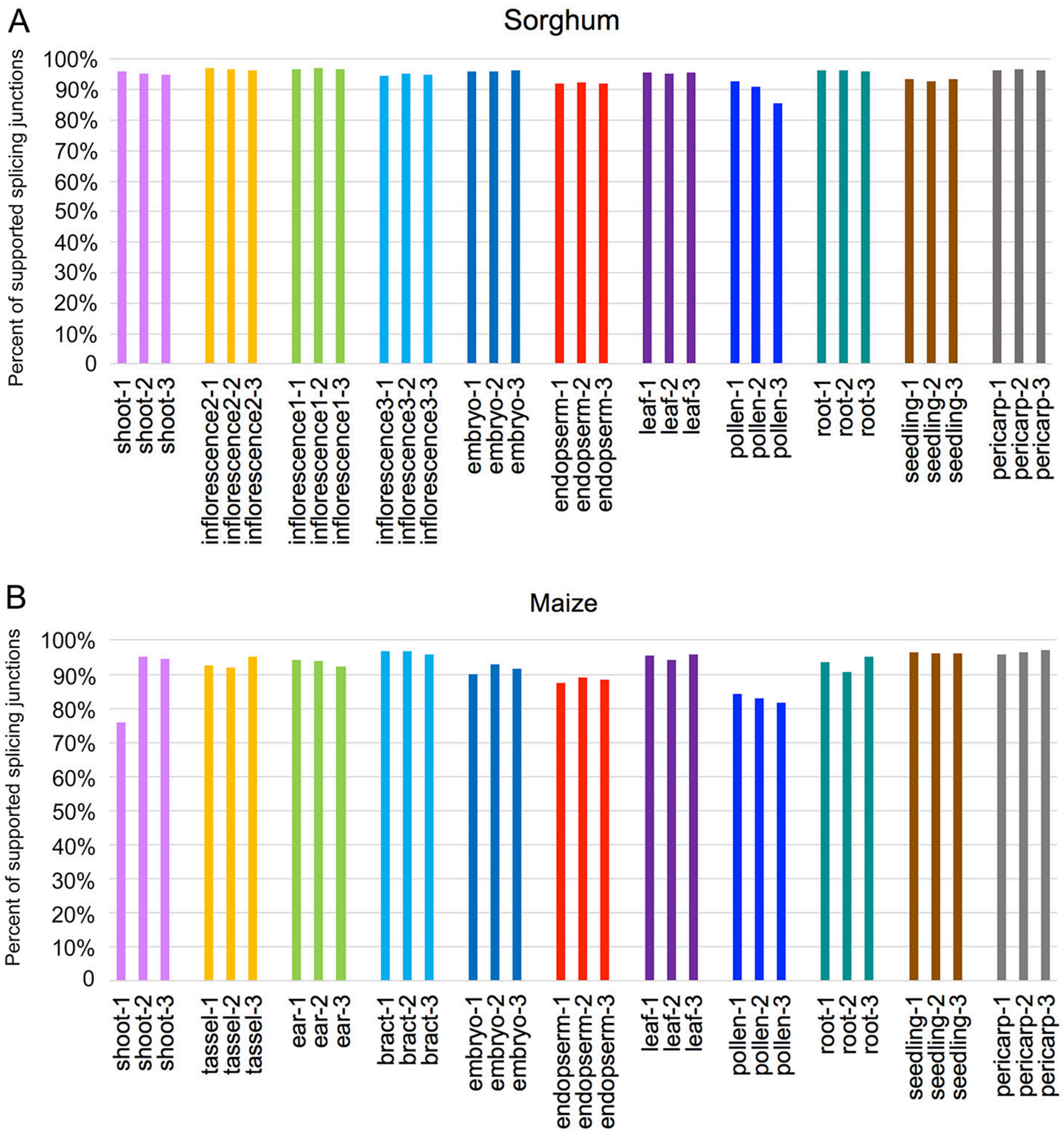

Supplemental Figure S1: Validation of PacBio isoforms by illumina short-reads sequencing. *(A)* Percent of supported PacBio isoforms of sorghum by short-reads sequencing. *(B)* Percent of supported PacBio isoforms of maize by short-reads sequencing.
