# Supplementary material for: A comparative transcriptional landscape of maize and sorghum obtained by single-molecule sequencing
Source: Genome Res. 2018 Jun;28(6):921–32. doi: 10.1101/gr.227462.117 (PMC5991521; doi:10.1101/gr.227462.117)
Supplement: Supplemental Material [file supp_gr.227462.117_Supplemental_Fig_S4.pdf]

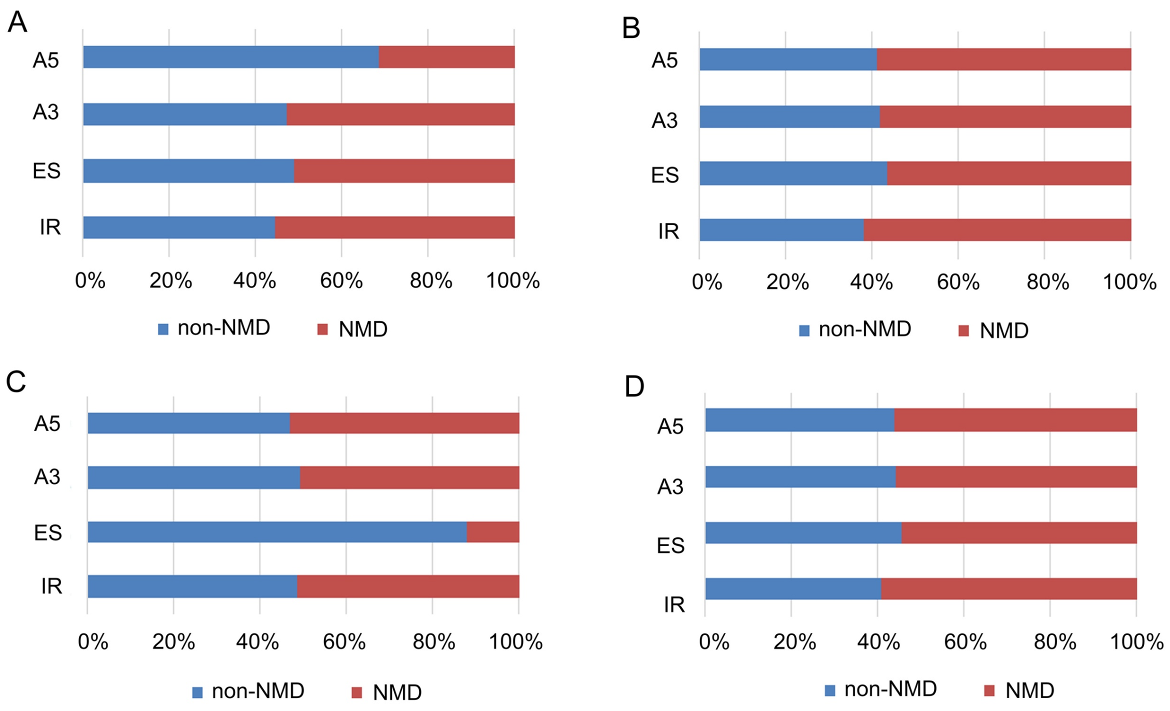

Supplemental Figure S4: AS events coupled with NMD.

(A) Proportion of NMD and non-NMD in sorghum alternative splicing events. *(B)* Proportion of NMD and non-NMD in maize alternative splicing events. *(C)* Distribution of NMD and non-NMD among conserved splicing events in sorghum. *(D)* Distribution of NMD and non-NMD among conserved splicing events in maize.
