# Supplementary material for: A comparative transcriptional landscape of maize and sorghum obtained by single-molecule sequencing
Source: Genome Res. 2018 Jun;28(6):921–32. doi: 10.1101/gr.227462.117 (PMC5991521; doi:10.1101/gr.227462.117)
Supplement: Supplemental Material [file supp_gr.227462.117_Supplemental_Fig_S5.pdf]

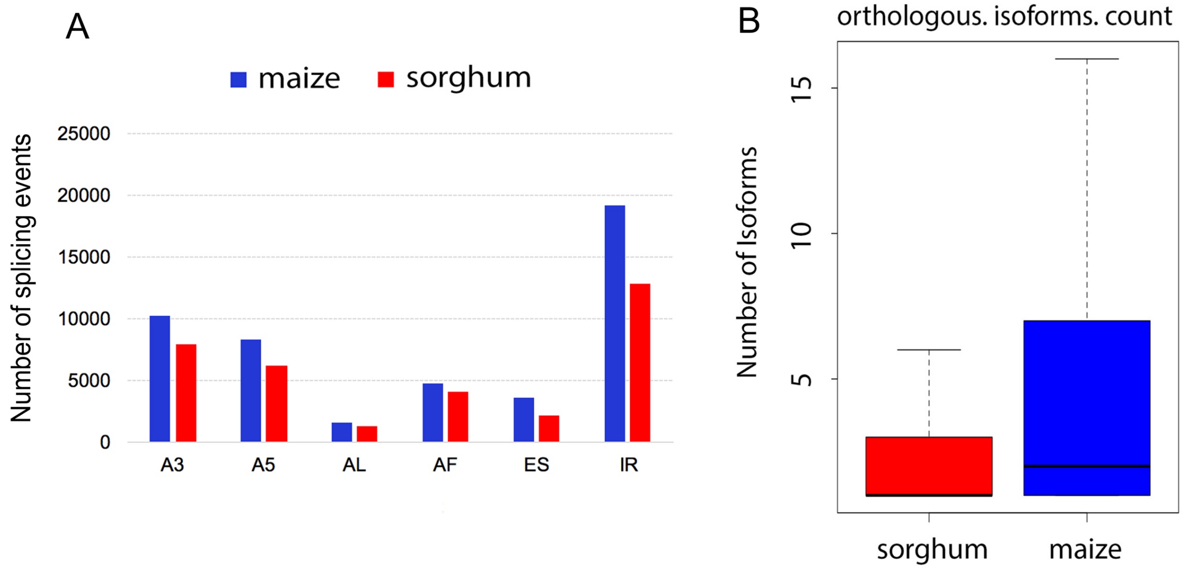

Supplemental Figure S5: Comparison of isoform number and splicing pattern between maize and sorghum orthologous genes.

(A) Splicing pattern distributions of maize and sorghum orthologous genes. *(B)* Number of isoforms per ortholog in maize and sorghum.
