# Supplementary material for: A comparative transcriptional landscape of maize and sorghum obtained by single-molecule sequencing
Source: Genome Res. 2018 Jun;28(6):921–32. doi: 10.1101/gr.227462.117 (PMC5991521; doi:10.1101/gr.227462.117)
Supplement: Supplemental Material [file supp_gr.227462.117_Supplemental_Fig_S8.pdf]

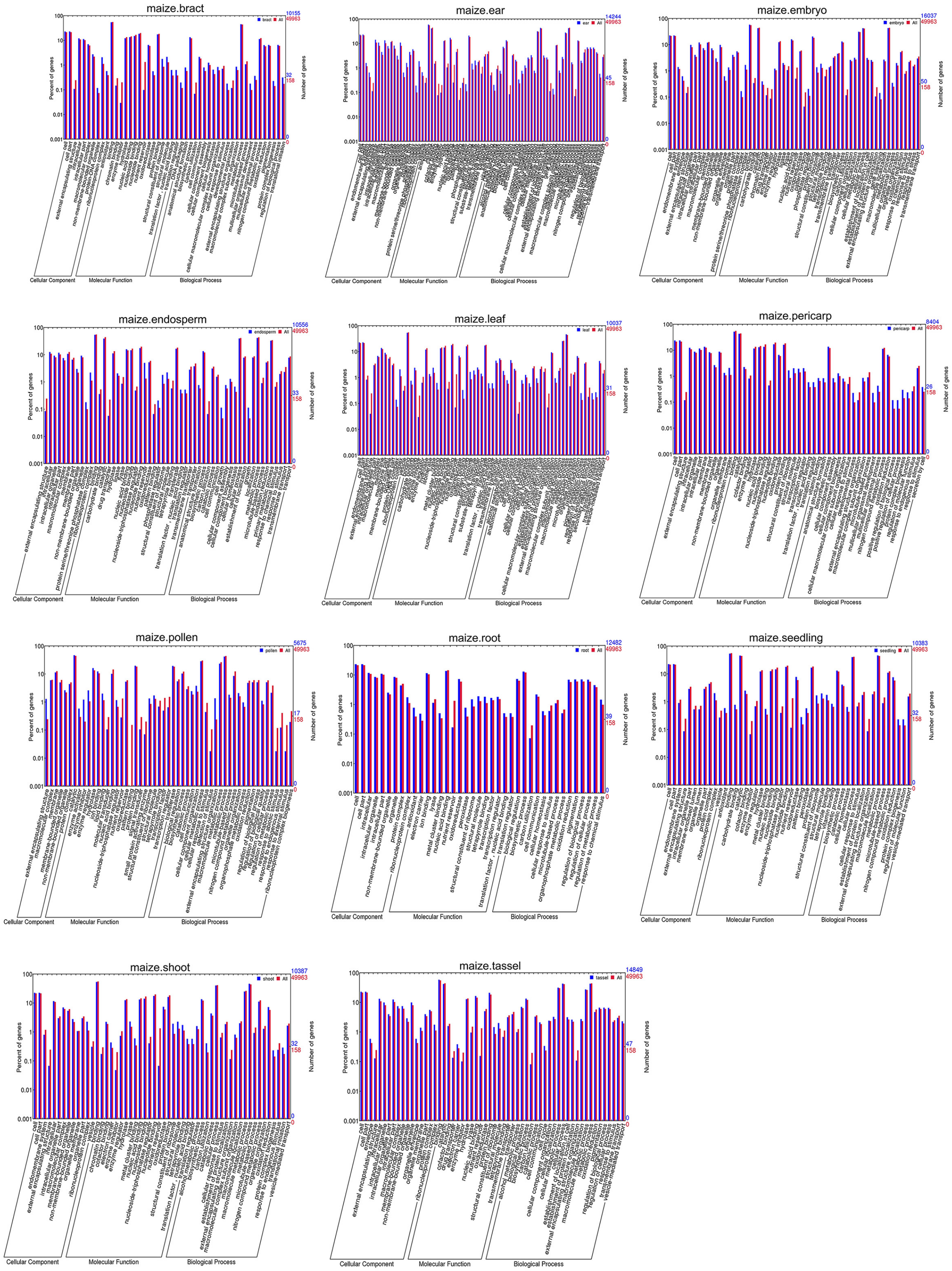
maize.bract
maize.ear
maize.embryo
maize.endosperm
maize.leaf
maize.pericarp
maize.pollen
maize.root
maize.seedling
maize.shoot
maize.tassel
Percent of genes
Number of genes
Cellular Component
Molecular Function
Biological Process

Supplemental Figure S8: Gene Ontology (GO) enrichment analysis of all isoforms among tissues in maize.
