# Supplementary material for: A comparative transcriptional landscape of maize and sorghum obtained by single-molecule sequencing
Source: Genome Res. 2018 Jun;28(6):921–32. doi: 10.1101/gr.227462.117 (PMC5991521; doi:10.1101/gr.227462.117)
Supplement: Supplemental Material [file supp_gr.227462.117_Supplemental_Fig_S10.pdf]

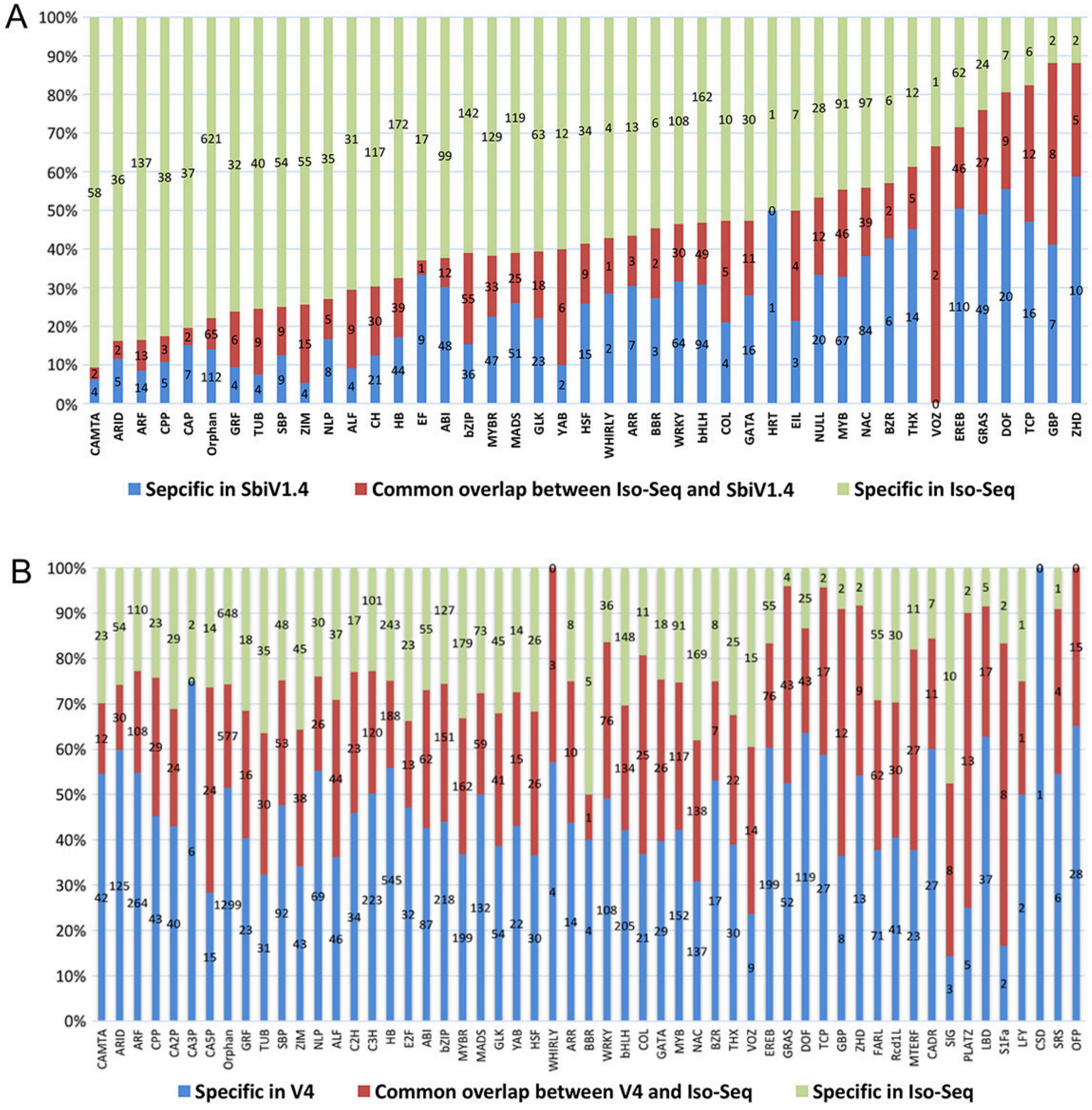

Supplemental Figure S10: Characterization of transcription factors in maize and sorghum.

(A) Characterization of transcription factors in sorghum. **(B)** Characterization of transcription factors in maize.
