# Supplementary material for: A comparative transcriptional landscape of maize and sorghum obtained by single-molecule sequencing
Source: Genome Res. 2018 Jun;28(6):921–32. doi: 10.1101/gr.227462.117 (PMC5991521; doi:10.1101/gr.227462.117)
Supplement: Supplemental Material [file supp_gr.227462.117_Supplemental_Fig_S18.pdf]

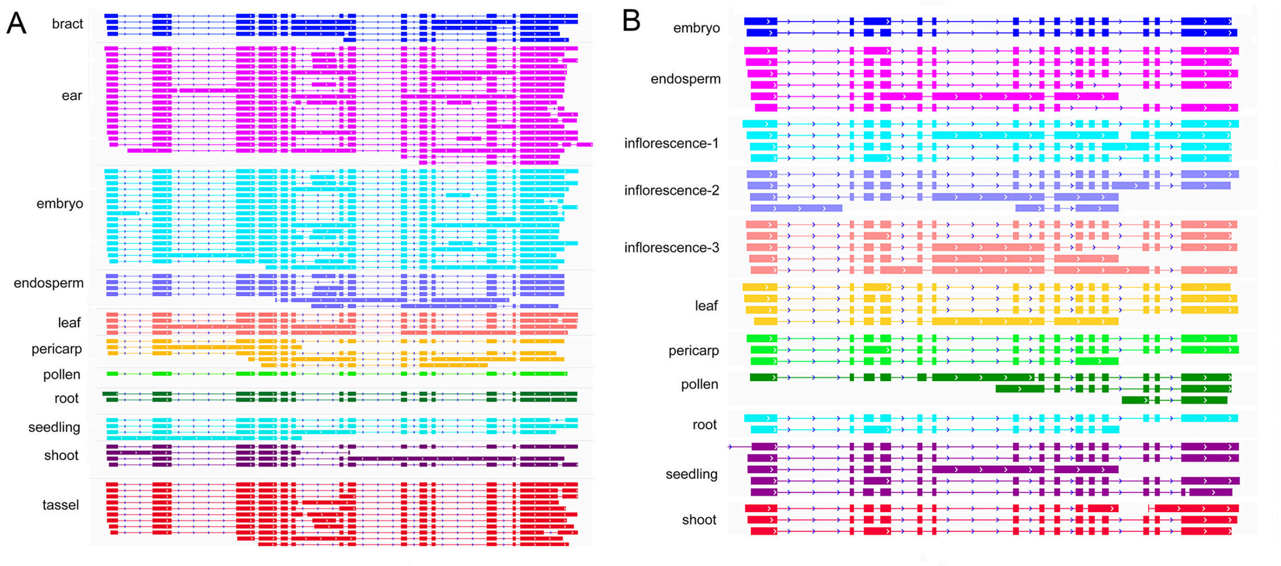


**Supplemental Figure S18: Example of different polyA cleavage sites in maize and sorghum isoforms.**

**(A)** Different polyA cleavage sites among isoforms in maize. **(B)** Different polyA cleavage sites exist among isoforms in sorghum.
