# Supplementary material for: A comparative transcriptional landscape of maize and sorghum obtained by single-molecule sequencing
Source: Genome Res. 2018 Jun;28(6):921–32. doi: 10.1101/gr.227462.117 (PMC5991521; doi:10.1101/gr.227462.117)
Supplement: Supplemental Material [file supp_gr.227462.117_Supplemental_Fig_S19.pdf]

APA motifs between maize and sorghum orthologous genes

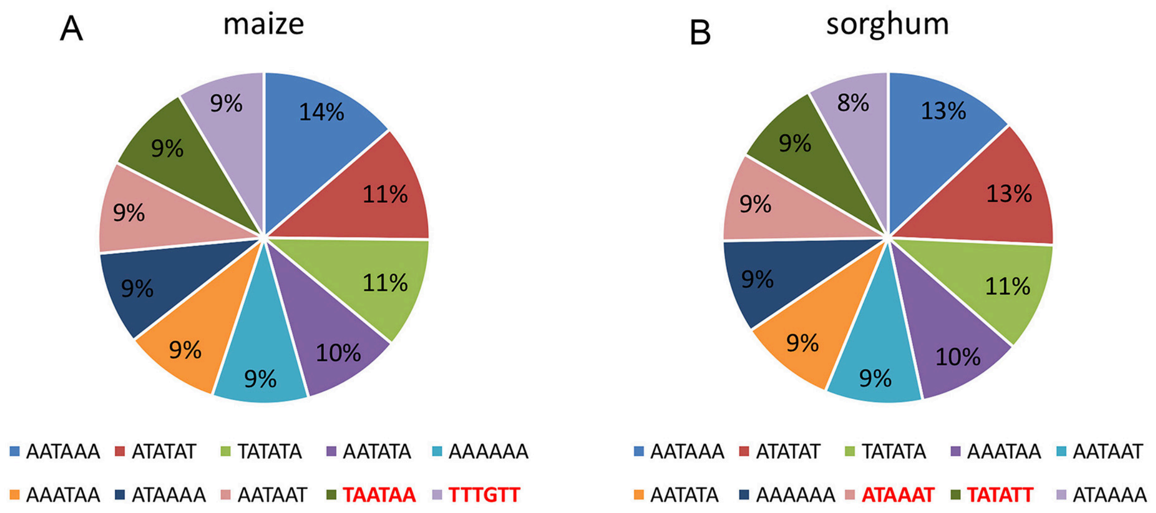

Supplemental Figure S19: APA motifs in maize and sorghum orthologous genes.

(A) Top10 APA motifs distribution in maize orthologous genes. **(B)** Top10 APA motifs distribution in sorghum orthologous genes.
