# Supplementary figures and images for: A comparative transcriptional landscape of maize and sorghum obtained by single-molecule sequencing
Source: Genome Res. 2018 Jun;28(6):921–32. doi: 10.1101/gr.227462.117 (PMC5991521; doi:10.1101/gr.227462.117)

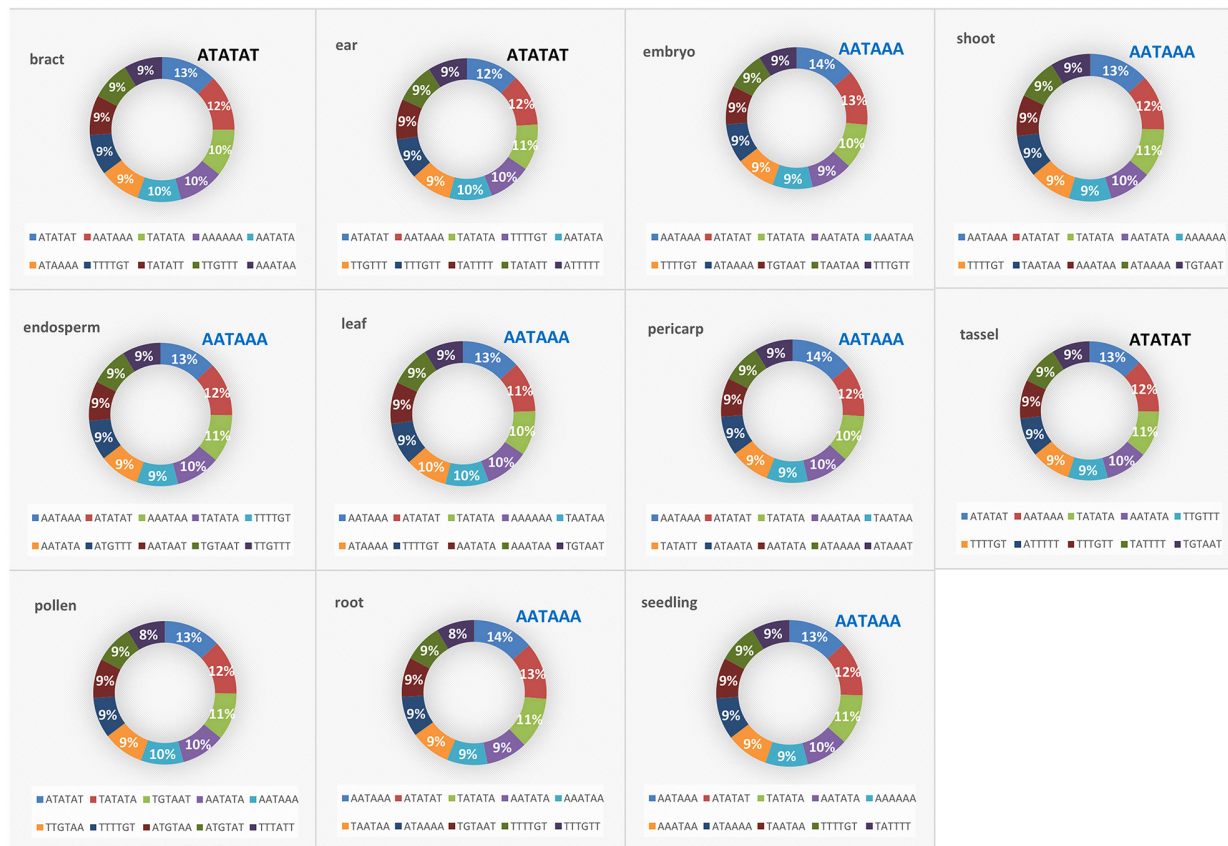

**Supplemental Figure S20: Distribution of top 10 APA motifs in maize isoforms across tissues.**

Supplement: Supplemental Material [file supp_gr.227462.117_Supplemental_Fig_S20.pdf]

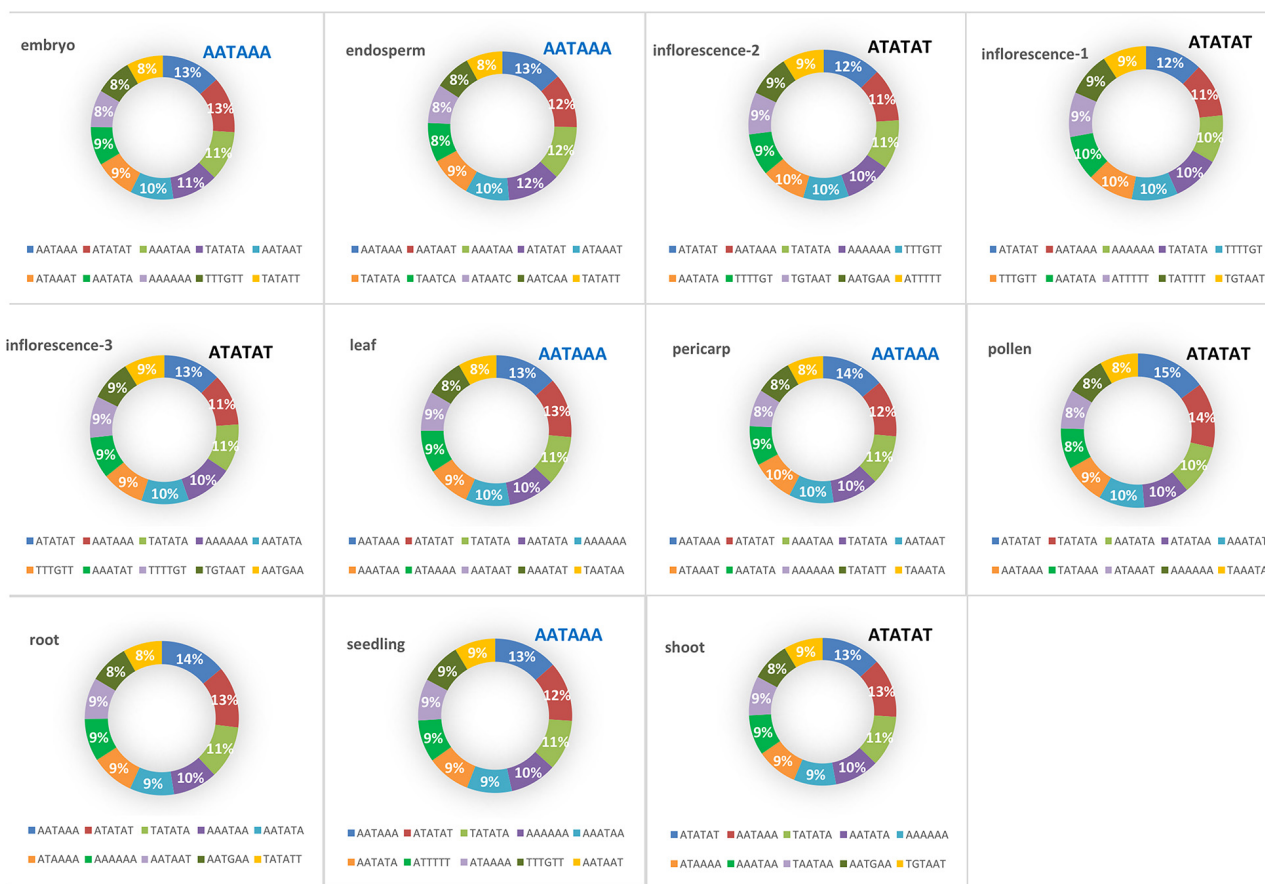

**Supplemental Figure S21: Distribution of top 10 APA motifs in sorghum isoforms across tissues.**

Supplement: Supplemental Material [file supp_gr.227462.117_Supplemental_Fig_S21.pdf]

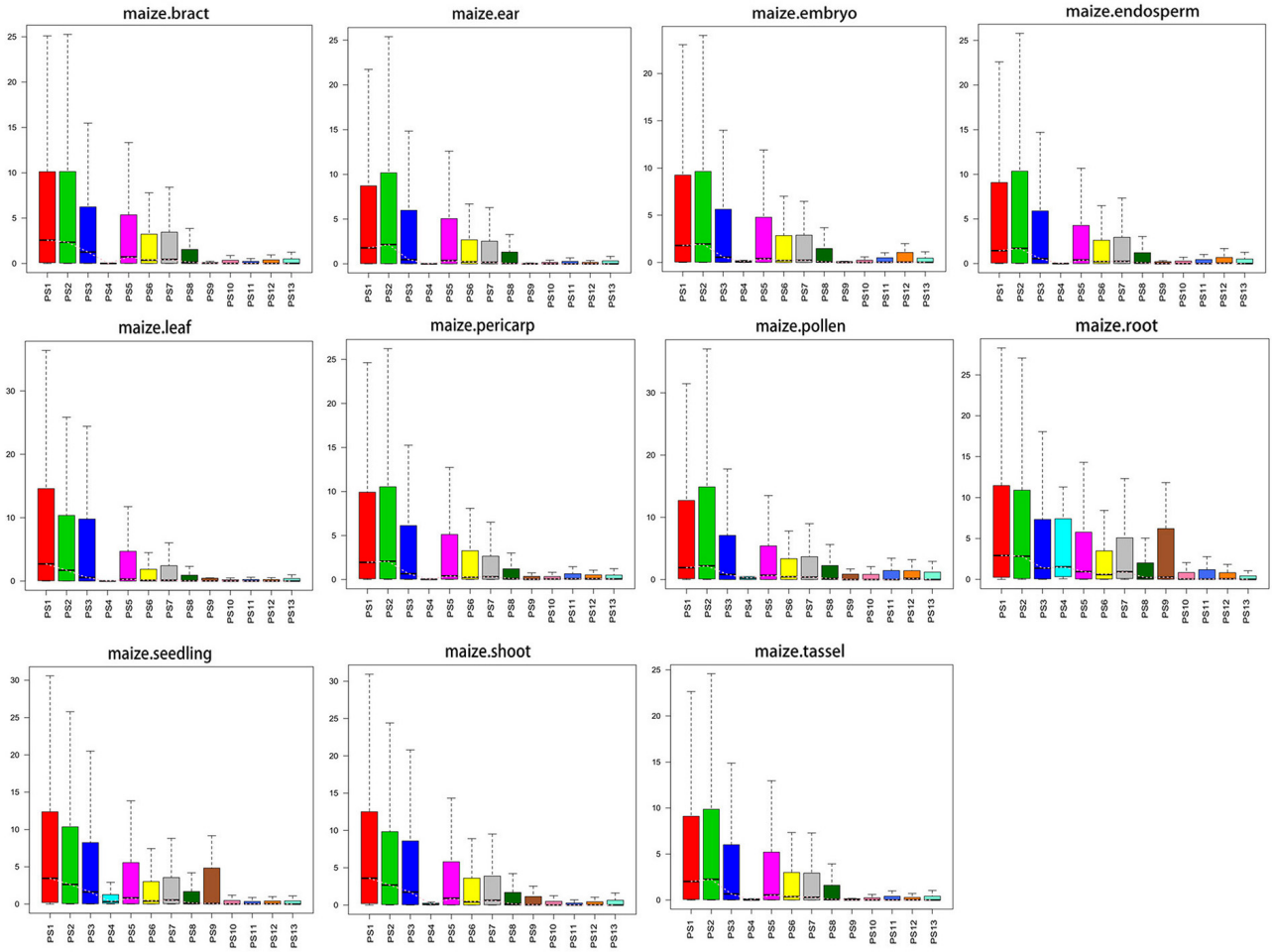

**Supplemental Figure S28: Gene expression in each tissue of maize across phylostratum (PS) ranks.**

Supplement: Supplemental Material [file supp_gr.227462.117_Supplemental_Fig_S28.pdf]

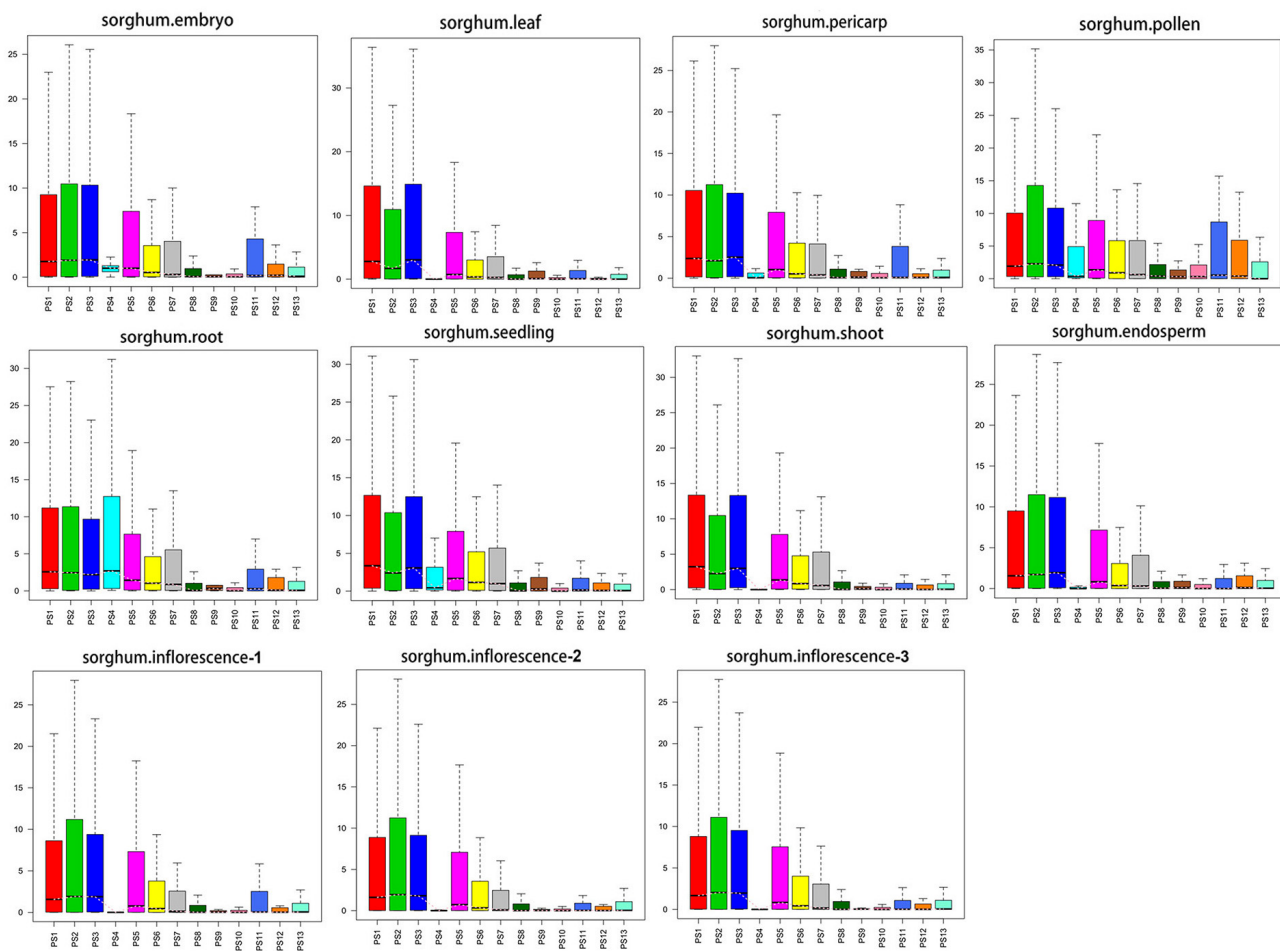

**Supplemental Figure S29: Gene expression in each tissue in of across phylostratum (PS) ranks.**

Supplement: Supplemental Material [file supp_gr.227462.117_Supplemental_Fig_S29.pdf]
