# Supplementary material for: A comparative transcriptional landscape of maize and sorghum obtained by single-molecule sequencing
Source: Genome Res. 2018 Jun;28(6):921–32. doi: 10.1101/gr.227462.117 (PMC5991521; doi:10.1101/gr.227462.117)
Supplement: Supplemental Material [file supp_gr.227462.117_Supplemental_Fig_S22.pdf]

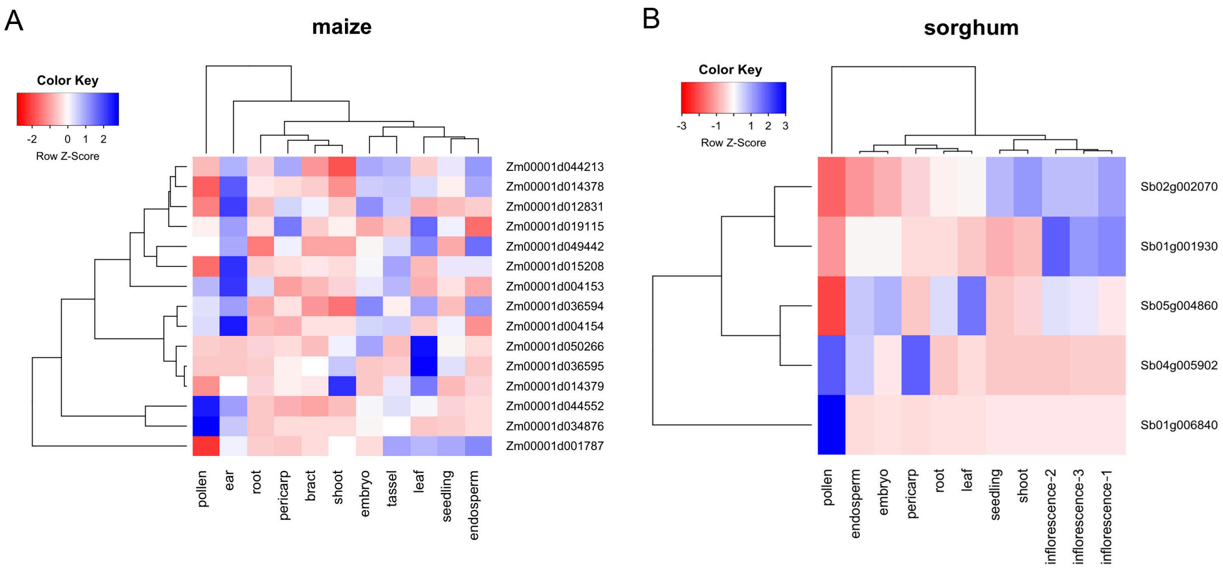

Supplemental Figure S22: Heatmap of polyadenylation machinery genes in maize and sorghum.

Heatmap of polyadenylation machinery genes in maize **(A)** and sorghum **(B)**.
