# Supplementary material for: A comparative transcriptional landscape of maize and sorghum obtained by single-molecule sequencing
Source: Genome Res. 2018 Jun;28(6):921–32. doi: 10.1101/gr.227462.117 (PMC5991521; doi:10.1101/gr.227462.117)
Supplement: Supplemental Material [file supp_gr.227462.117_Supplemental_Fig_S25.pdf]

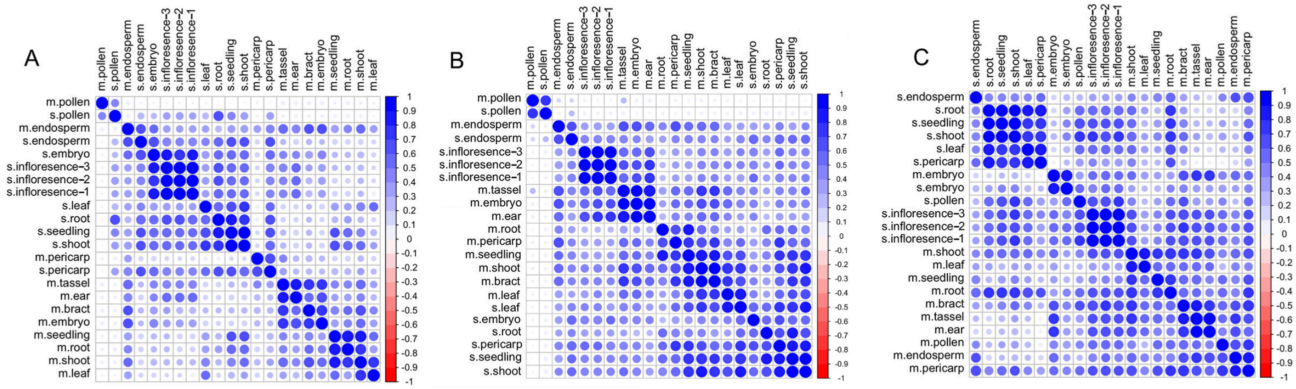

Supplemental Figure S25: Correlation matrix of TFs in maize A genome only, B genome only, and shared by A and B genome.

Correlation matrix of: **(A)** TFs expression among tissues of maize A genome uniq TFs. **(B)** TFs expression among tissues of maize B genome uniq TFs. **(C)** TFs expression among tissues in shared TFs between maize A and B genome.
