# Supplementary material for: A comparative transcriptional landscape of maize and sorghum obtained by single-molecule sequencing
Source: Genome Res. 2018 Jun;28(6):921–32. doi: 10.1101/gr.227462.117 (PMC5991521; doi:10.1101/gr.227462.117)
Supplement: Supplemental Material [file supp_gr.227462.117_Supplemental_Fig_S26.pdf]

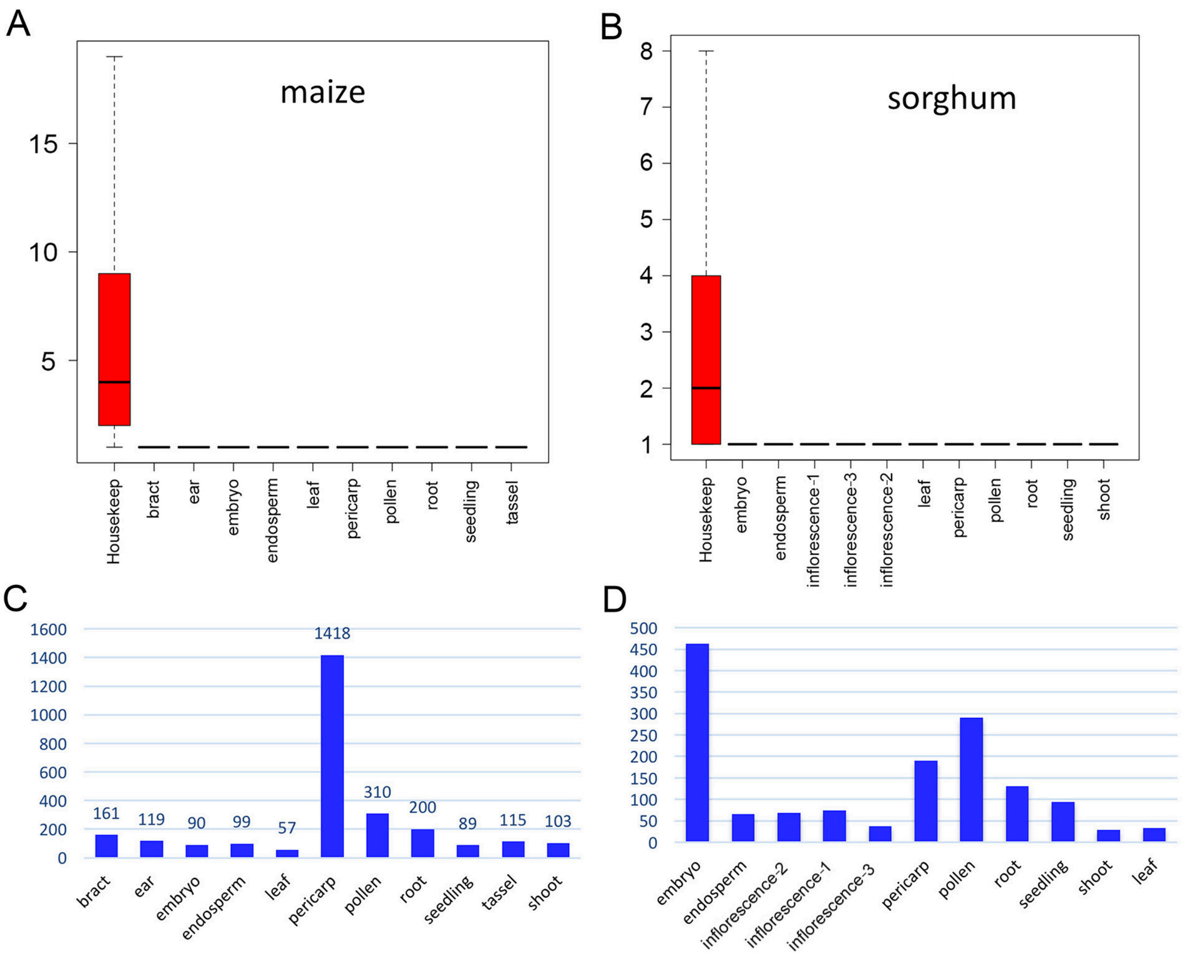

Supplemental Figure S26: Characteristics of housekeeping genes and tissue specific genes.

(A) Comparison of number of isoforms between housekeeping genes and tissue specific genes in maize. **(B)** Comparison of number of isoforms between housekeeping genes and tissue-specific genes in sorghum. **(C)** Number of tissue specific genes in each tissue in maize. **(D)** Number of tissue specific genes in each tissue in sorghum.
