# Supplementary material for: A comparative transcriptional landscape of maize and sorghum obtained by single-molecule sequencing
Source: Genome Res. 2018 Jun;28(6):921–32. doi: 10.1101/gr.227462.117 (PMC5991521; doi:10.1101/gr.227462.117)
Supplement: Supplemental Material [file supp_gr.227462.117_Supplemental_Table_S1.pdf]

Supplemental Table 1. Sequencing summary of size-fractionated libraries.

Sample	Cells	Total reads*	Total full-length reads	% full-length Reads
< 1kb	13	533,437	259,904	48.4%
1–2 kb	13	953,299	594,588	62.1%
2–3 kb	13	840,463	505,045	59.96%
3–5 kb	13	457,863	148,885	32.2%
> 5 kb	13	638,475	81,011	10.3%
< 1kb	13	552,393	288,878	51.4%
1–2 kb	13	931,305	615,194	65.3%
2–3 kb	13	626,482	341,189	54.2%
3–5 kb	13	671,458	228,516	33.9%
> 5 kb	13	688,105	73,038	10.3%
