# Supplementary material for: A comparative transcriptional landscape of maize and sorghum obtained by single-molecule sequencing
Source: Genome Res. 2018 Jun;28(6):921–32. doi: 10.1101/gr.227462.117 (PMC5991521; doi:10.1101/gr.227462.117)
Supplement: Supplemental Material [file supp_gr.227462.117_Supplemental_Table_S2.pdf]

Supplemental Table 2. Number of high quality (HQ) and low quality (LQ) transcripts after processing.

Tissue	Species	HQ transcripts	LQ transcripts
silk	maize	270	106
bract	maize	85,697	8,329
shoot	maize	93,860	9,019
seedling	maize	76,479	7,267
leaf	maize	135,576	18,992
pericarp	maize	72,882	6,777
seedling	sorghum	149,766	15,556
shoot	sorghum	72,929	6,740
endopserm	sorghum	156,569	32,964
root	sorghum	74,748	6,419
pollen	sorghum	84,200	23,893
leaf	sorghum	93,938	10,281
pericarp	sorghum	93,263	13,066
Infloresence-1	sorghum	87,320	12,890
Infloresence-2	sorghum	67,811	4,940
Infloresence-3	sorghum	126,507	10,985
embryo	sorghum	79,827	8,404
root tip	sorghum	72,434	5,734
**Total**	**m/s**	**1,624,076**	**202,362**
