# Supplementary material for: A comparative transcriptional landscape of maize and sorghum obtained by single-molecule sequencing
Source: Genome Res. 2018 Jun;28(6):921–32. doi: 10.1101/gr.227462.117 (PMC5991521; doi:10.1101/gr.227462.117)
Supplement: Supplemental Material [file supp_gr.227462.117_Supplemental_Table_S3.pdf]

Supplemental Table 3. Conserved lncRNAs between maize and sorghum.

Sorghum	Maize	%identity	alignment length	mismatches	gap openings	q.start	q.end	s.start	s.end	e-value	bit score
PB.22291.1|GL004789.1:757-2685(-)|i1HQ_root|c112445/f4p28/1831	PB.13336.1:chr4:115008091-115015269(-):i0HQ_embryo:c24092/f1p54/1648	98.78	1643	17	3	4	1643	3	1645	0	3075
PB.10312.23|chr3:61453738-61486976(-)|i0HQ_seedling|c12157/f1p109/686	PB.11619.10:chr3:202063960-202064907(-):i0HQ_root:c17643/f1p44/949	90.14	507	31	7	9	512	10	500	1.00E-165	577
PB.22057.4|GL002653.1:12002-14069(-)|i1HQ_infloresence3|c13026/f1p39/1350	PB.221.1:chr1:8183217-8186717(+):i2HQ_endosperm:c9110/f1p11/3520	96.05	1140	32	5	6	1145	3016	1890	0	1879
PB.10312.22|chr3:61453738-61454433(-)|i0HQ_infloresence3|c4438/f1p157/702	PB.11619.10:chr3:202063960-202064907(-):i0HQ_root:c17643/f1p44/949	88.27	520	38	7	7	526	4	500	1.00E-149	523
PB.22057.2|GL002653.1:11920-14069(-)|i1HQ_leaf|c9610/f1p42/1327	PB.221.1:chr1:8183217-8186717(+):i2HQ_endosperm:c9110/f1p11/3520	96.73	1009	30	3	5	1013	3016	2011	0	1715
