# Supplementary material for: A comparative transcriptional landscape of maize and sorghum obtained by single-molecule sequencing
Source: Genome Res. 2018 Jun;28(6):921–32. doi: 10.1101/gr.227462.117 (PMC5991521; doi:10.1101/gr.227462.117)
Supplement: Supplemental Material [file supp_gr.227462.117_Supplemental_Table_S4.pdf]

Supplemental Table 4. 16-mer barcode sequences used for PacBio barcoding libraries.

Oligo	Sequence
dT_BC1	AAGCAGTGGTATCAACGCAGAGTACTCAGACGATGCGTCATTTTTTTTTTTTTTTTTTTTTTTTTTTTTTTVN
dT_BC2	AAGCAGTGGTATCAACGCAGAGTACCTATACATGACTCTGCTTTTTTTTTTTTTTTTTTTTTTTTTTTTTTVN
dT_BC3	AAGCAGTGGTATCAACGCAGAGTACTACTAGAGTAGCACTC TTTTTTTTTTTTTTTTTTTTTTTTTTTTTTVN
dT_BC4	AAGCAGTGGTATCAACGCAGAGTACTGTGTATCAGTACATGTTTTTTTTTTTTTTTTTTTTTTTTTTTTTTVN
dT_BC5	AAGCAGTGGTATCAACGCAGAGTACGATCTCTACTATATGCTTTTTTTTTTTTTTTTTTTTTTTTTTTTTTVN
dT_BC6	AAGCAGTGGTATCAACGCAGAGTACACAGTCTATACTGCTGTTTTTTTTTTTTTTTTTTTTTTTTTTTTTTVN
dT_BC7	AAGCAGTGGTATCAACGCAGAGTACCATAGCGACTATCGTGTTTTTTTTTTTTTTTTTTTTTTTTTTTTTTVN
dT_BC8	AAGCAGTGGTATCAACGCAGAGTACCGAGCACGCGCGTGTGTTTTTTTTTTTTTTTTTTTTTTTTTTTTTTVN
dT_BC9	AAGCAGTGGTATCAACGCAGAGTACGCTCGACTGTGAGAGATTTTTTTTTTTTTTTTTTTTTTTTTTTTTTVN
