# Supplementary material for: A comparative transcriptional landscape of maize and sorghum obtained by single-molecule sequencing
Source: Genome Res. 2018 Jun;28(6):921–32. doi: 10.1101/gr.227462.117 (PMC5991521; doi:10.1101/gr.227462.117)
Supplement: Supplemental Material [file supp_gr.227462.117_Supplemental_Fig_S12.pdf]

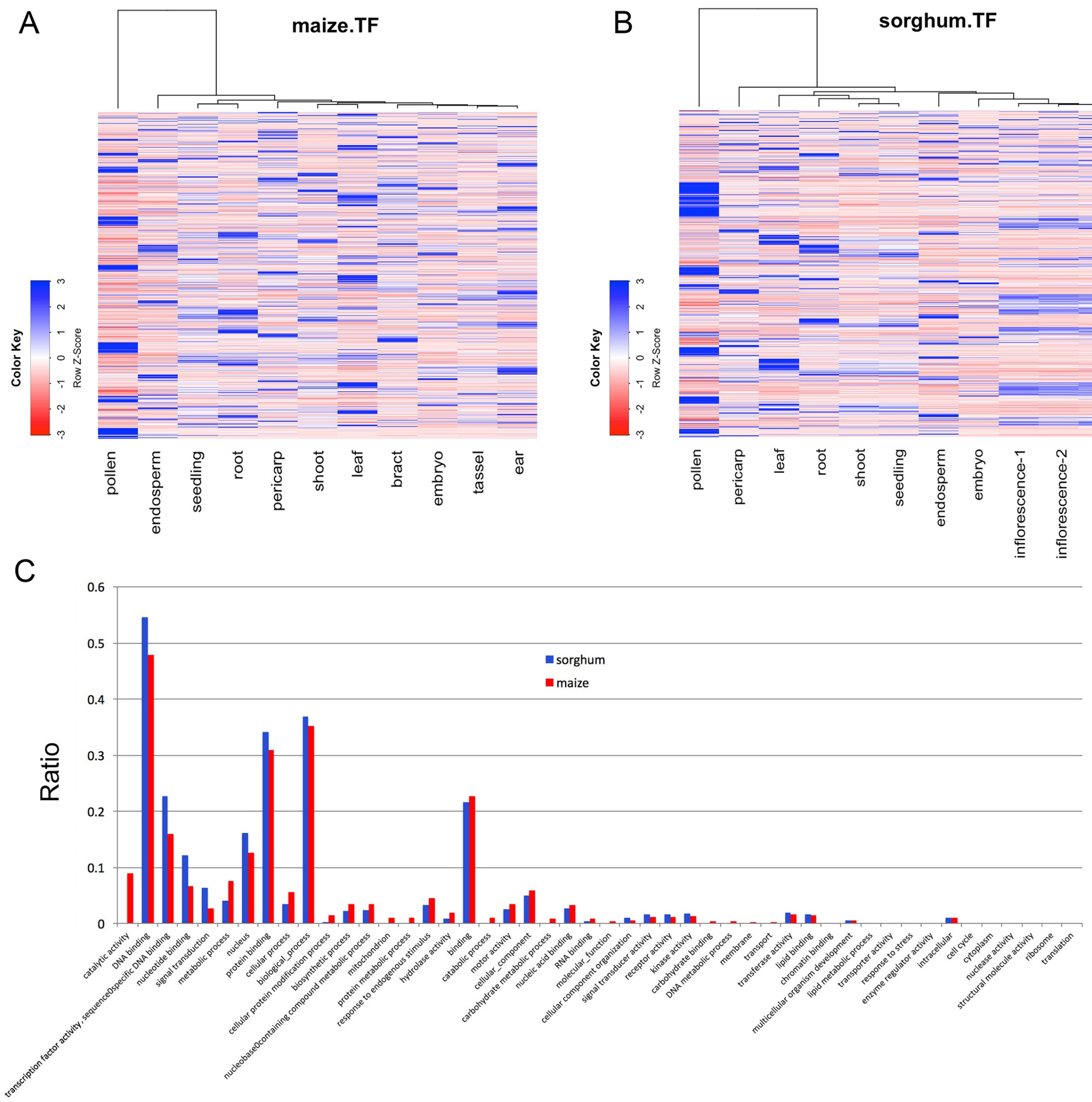

Supplemental Figure S12: Expression of transcription factors in maize and sorghum among different tissues and comparison of gene ontology enrichment. **(A)** Expression of different TFs among different tissues in maize. **(B)** Expression of TFs among different tissues in sorghum. **(C)** Gene ontology enrichment between maize and sorghum TFs.
