# Supplementary material for: A comparative transcriptional landscape of maize and sorghum obtained by single-molecule sequencing
Source: Genome Res. 2018 Jun;28(6):921–32. doi: 10.1101/gr.227462.117 (PMC5991521; doi:10.1101/gr.227462.117)
Supplement: Supplemental Material [file supp_gr.227462.117_Supplemental_Methods.pdf]

Supplemental Methods

RNA preparation

Total RNA was prepared by grinding tissue in TRIzol reagent (Invitrogen 15596026) on dry ice and processing as recommended by the manufacturer. To remove DNA, an aliquot of total RNA was treated with RQ1 DNase (Promega M6101), followed by phenol/chloroform/isoamyl alcohol extraction, chloroform/isoamyl alcohol extraction using Phase Lock Gel Light tubes (5 PRIME 2302800), and ethanol precipitation. Precipitated RNA was stored at -20°C.

Illumina RNA-seq library construction

Total RNA (20 μg) was used for poly(A)$^+$ selection using oligo(dT) magnetic beads (Invitrogen 610-02), eluted in water, and subjected to RNA-seq library construction using the ScriptSeq™ kit (Epicentre SS10906). Libraries were amplified by 15 cycles of PCR, and then sequenced in two lanes on the HiSeq 2500 PE125 platform at Woodbury Genome Center, Cold Spring Harbor Laboratory.

Barcoding library and single-molecule sequencing

Tissue-specific 16-mer barcodes were added before pooling for subsequent amplification (Supplementary Table S4). To avoid loading bias, which favors sequencing of shorter transcripts, multiple size-fractionated libraries (<1 kb, 1–2 kb, 2–3 kb, 3–5 kb, and >5 kb) were constructed using a SageELF device, as described (Wang et al. 2016). Barcoded SMRTBell libraries were sequenced on a PacBio RS II with 130 SMRT Cells and a PacBio Sequel with five cells using P6–C4 chemistry.

Illumina data analysis

Raw reads were aligned to the B73 reference genome (RefGen_v4) (Jiao et al. 2017) and sorghum BTX623 reference genome Sbi1.4 (Paterson et al. 2009) using STAR (Dobin et al. 2013) with minimum intron length set to 20 bp and maximum intron length set to 50 kb, with default settings for other parameters.

Mapping of PacBio data

The PacBio raw reads generated from the RSII platform were classified into Circular Consensus Sequences (CCS) and non-CCS subreads using ToFu (Wang et al. 2016) by searching for the presence of sequencing adapters. ToFU determined a CCS or subread sequence to be full-length if both the 5' and 3' cDNA primers were present and a poly(A) tail signal preceded the 3' primer. To improve consensus accuracy, we used the isoform-level clustering algorithm ICE (Iterative Clustering for Error Correction), and polished the full-length consensus sequences from ICE using Quiver. This method generated full-length transcripts with ≥ 99% post-correction accuracy. Mapping of full-length CCS reads to the maize RefGen_v4 genome assembly (Jiao et al. 2017) and sorghum Sbi1.4 genome assembly (Paterson et al. 2009) was carried out using GMAP (Wu et al. 2005). Mapped full-length reads were further aligned by BLASTN to all NCBI RefSeq to consolidate the confidence, and then collapsed using the pbtranscript-ToFU package (http://github.com/PacificBiosciences/cDNA_primer/) with min-coverage = 85% and min-identity = 90% to collapse redundant transcripts; however, the 5' difference was not considered when collapsing the reads. Each isoform was compared with existing gene models of RefGen_v4 annotation using

gffcompare (Pertea et al. 2016), and the isoforms were further classified into eight groups based on their exon structures (splicing junctions).

For the Sequel runs, the five cells were combined and run through the SMRTLink (SMRTAnalysis 2.3) Iso-Seq pipeline, the officially implemented version of ToFU. The ToFU version used to process the RS2 data and Iso-Seq SA3.2, used to process the Sequel data, are algorithmically identical, with differences only in input movie format (.bax.h5 HDF5 format for RS2 and .subreads.bam BAM format for Sequel).

lncRNA identification from PacBio sequences

The 1,704 known high-confidence lncRNAs (Li et al. 2014) and 867 lncRNAs (Wang et al. 2016) recently identified by single-molecule sequencing were used to build a model for scanning maize data using PLEK (Li et al. 2014). Recently identified sorghum lncRNAs were used for PLEK modeling to scan the sorghum data for novel lncRNA identification. All PacBio isoforms were predicted based on the model, and ORFs of candidate lncRNAs were predicted using EMBOSS. Transcripts encoding ORFs longer than 100 amino acids were filtered out, and the remaining transcripts were further screened by BLASTX (e-value $\leq 1.0 \times 10^{-10}$) against protein sequences of rice, *Brachypodium,* and sorghum, downloaded from Gramene release b46 (Tello-Ruiz et al., 2016) (http://ensembl.gramene.org/info/website/ftp/index.html).

Functional annotation of PacBio isoforms

To investigate the functions of the isoforms in each tissue, InterProScan-5.16-55.0 (Mitchell et al. 2015) (using default settings) was run to map known protein domains to all isoforms. Using InterPro2GO mapping, GO terms were obtained for the InterPro domains. WEGO (Ye et al. 2006)

was used to determine the enrichment of GO terms in each tissue in comparison with all tissues under Pearson Chi-Square test.

Supplemental References

Paterson AH, Bowers JE, Bruggmann R, Dubchak I, Grimwood J, Gundlach H, Haberer G, Hellsten U, Mitros T, Poliakov A, et al. 2009. The Sorghum bicolor genome and the diversification of grasses. *Nature* **457**: 551-6.

Wu TD, Watanabe CK. 2005. GMAP: a genomic mapping and alignment program for mRNA and EST sequences. *Bioinformatics* **21**: 1859-75.

Dobin A, Davis CA, Schlesinger F, Drenkow J, Zaleski C, Jha S, Batut P, Chaisson M, Gingeras TR. 2013. STAR: ultrafast universal RNA-seq aligner. *Bioinformatics* **29**, 15-21.

Pertea M, Kim D, Pertea GM, Leek JT, Salzberg SL. 2016. Transcript-level expression analysis of RNA-seq experiments with HISAT, StringTie and Ballgown. *Nat Protoc* **11**: 1650-67.

Mitchell A, Chang HY, Daugherty L, Fraser M, Hunter S, Lopez R, McAnulla C, McMenamin C, Nuka G, Pesseat S et al. 2015. The InterPro protein families database: the classification resource after 15 years. *Nucleic Acids Res* **43**, D213-21.

Ye J, Fang L, Zheng H, Zhang Y, Chen J, Zhang Z, Wang J, Li S, Li R, Bolund L, et al. 2006. WEGO: a web tool for plotting GO annotations. *Nucleic Acids Res* **34**, W293-7.
